# Regulation of NF-κB Oscillation by Nuclear Transport: Mechanisms Determining the Persistency and Frequency of Oscillation

**DOI:** 10.1371/journal.pone.0127633

**Published:** 2015-06-04

**Authors:** Daisuke Ohshima, Kazuhisa Ichikawa

**Affiliations:** Division of Mathematical Oncology, The Institute of Medical Science, The University of Tokyo, Minato-ku, Tokyo, Japan; University of Toronto, CANADA

## Abstract

The activated transcription factor NF-κB shuttles between the cytoplasm and the nucleus resulting in the oscillation of nuclear NF-κB (NF-κB_n_). The oscillation pattern of NF-κB_n_ is implicated in the regulation of gene expression profiles. Using computational models, we previously reported that spatial parameters, such as the diffusion coefficient, nuclear to cytoplasmic volume ratio, transport through the nuclear envelope, and the loci of translation of IκB protein, modified the oscillation pattern of NF-κB_n_. In a subsequent report, we elucidated the importance of the “reset” of NF-κB_n_ (returning of NF-κB to the original level) and of a “reservoir” of IκB in the cytoplasm. When the diffusion coefficient of IκB was large, IκB stored at a distant location from the nucleus diffused back to the nucleus and “reset” NF-κB_n_. Herein, we report mechanisms that regulate the persistency and frequency of NF-κB_n_ oscillation by nuclear transport. Among the four parameters of nuclear transport tested in our spatio-temporal computational model, the export of IκB mRNA from the nucleus regulated the persistency of oscillation. The import of IκB to the nucleus regulated the frequency of oscillation. The remaining two parameters, import and export of NF-κB to and from the nucleus, had virtually no effect on the persistency or frequency. Our analyses revealed that lesser export of IκB mRNA allowed NF-κB_n_ to transcript greater amounts of IκB mRNA, which was retained in the nucleus, and was subsequently exported to the cytoplasm, where large amounts of IκB were synthesized to “reset” NF-κB_n_ and drove the persistent oscillation. On the other hand, import of greater amounts of IκB led to an increase in the influx and the efflux of NF-κB to and from the nucleus, resulting in an increase in the oscillation frequency. Our study revealed the importance of nuclear transport in regulating the oscillation pattern of NF-κB_n_.

## Introduction

NF-κB is a transcription factor that regulates the expression profiles of a vast number of genes. In the classical pathway of NF-κB activation, extracellular stimuli such as TNFα lead to the phosphorylation and proteasomal degradation of IκB, a negative regulator of NF-κB, which retains it in the cytoplasm in the resting state. NF-κB thus liberated from inhibition translocates to the nucleus, leading to the expression of genes, including IκB. Newly synthesized IκB protein binds to NF-κB in the nucleus causing an export of nuclear NF-κB (NF-κB_n_), leading to the raise of the cytoplasmic concentration of NF-κB again. If the NF-κB activating stimulus remains, IκB in the IκB:NF-κB complex is degraded, and the liberated NF-κB translocates to the nucleus again. Thus, the oscillation of NF-κB emerges [1–6].

The biological functions of NF-κB oscillation and its mechanisms have been investigated by multiple research groups leading to the discovery of several sensitive parameters affecting of NF-κB oscillation [7–14], the importance of the transport of proteins, including NF-κB [15], importance of negative feedback and inhibitor proteins [16–19], the effects of stimulation pattern on gene expression profiles [5,20] and stimulus specificity of gene expression [21]. These analyses revealed the critical parameters that regulate the activity of NF-κB, including negative feedback loops, rate constants, concentrations of molecules, and patterns of stimulation. Previously, we constructed a three-dimensional (3D) spatio-temporal model of NF-κB oscillation, and reported the importance of spatial parameters in the regulation of the NF-κB_n_ oscillation pattern [22]. We found several factors, such as the diffusion coefficient, nuclear transport, nuclear to cytoplasmic volume (N/C) ratio, and loci of protein synthesis, to regulate the oscillation pattern of NF-κB_n_. In a subsequent report, we showed why the diffusion coefficient regulated the oscillation pattern [23]. We revealed that the “reset” of NF-κB_n_ was critical for the sustained oscillation of NF-κB_n_, and that a distant location in the cytoplasm acted as a “reservoir” for newly synthesized IκB, which subsequently caused the “reset” of NF-κB_n_. A larger diffusion coefficient of IκB helped to store a greater amount of IκB in the cytoplasm, which could then diffuse back to the nucleus to sufficiently “reset” NF-κB_n_. Furthermore, we identified a possible change in the effective value of the diffusion coefficient caused by a change in the crowdedness of organelles, which was observed in hypoxic cells [24].

Herein, we report how and why nuclear transport regulates the oscillation pattern of NF-κB_n_. Several reports are available regarding the effect of nuclear transport on the activity of NF-κB in the nucleus. An earlier study showed that an siRNA-induced knock-down of nucleoporin Nup88, which is one of the constituent proteins of the nuclear pore complex (NPC) at the cytoplasmic face, prevented the nuclear accumulation of NF-κB and reduced the expression of a reporter gene [25]. Senescence has been shown to alter the expression of nucleoporins (nups) and to decrease the number of NPCs, thus impairing the nuclear translocation of NF-κB [26]. In addition, leukemogenic Nup98 fusion proteins have been reported to cause aberrant localization of CRM1, which is required for the export of NF-κB_n_, and the nuclear accumulation of NF-κB [27]. This was correlated with the enhanced transcription activity of NF-κB_n_. Constitutively active NF-κB was found in malignant melanoma cells, and knock-down of the *Nup88* gene reduced the nuclear accumulation of NF-κB in these cells [25]. Aberrant localization of CRM1 caused by over expression of Nup88 and Nup214 has also been reported [28]. Although all these reports strongly suggested the importance of nuclear transport in the regulation of NF-κB, it is possible that the reduced or increased localization of NF-κB in the nucleus resulting due to the reduced or increased nuclear transport could be a simple causal effect, while there may exist a much more complicated mechanisms for the regulation of NF-κB_n_ by nuclear transport. To elucidate the effect of alterations in parameters affecting the nuclear transport on the oscillation pattern of NF-κB, we conducted spatio-temporal simulations.

We investigated four pathways of nuclear transport in our spatio-temporal model: NF-κB import to the nucleus, its export from the nucleus, export of IκB mRNA (mRNA_IκB_) from the nucleus, and the import of newly synthesized IκB to the nucleus. We performed simulations by modifying each one of these parameters, and found that these four nuclear transport pathways altered the oscillation pattern of NF-κB_n_ differently. While alteration in NF-κB nuclear import and its export from the nucleus resulted in no appreciable change in the oscillation pattern, alteration in the nuclear export of mRNA_IκB_ and the import of IκB altered the persistency and the frequency of oscillation, respectively. Furthermore, reduction but not enhancement of the nuclear export of mRNA_IκB_ increased the persistency of the oscillation, which was an unexpected result.

## Results

### Export of mRNA_IκB_ from and import of IκB to the nucleus independently regulate persistency and frequency of NF-κB oscillation

**Fig 1:**
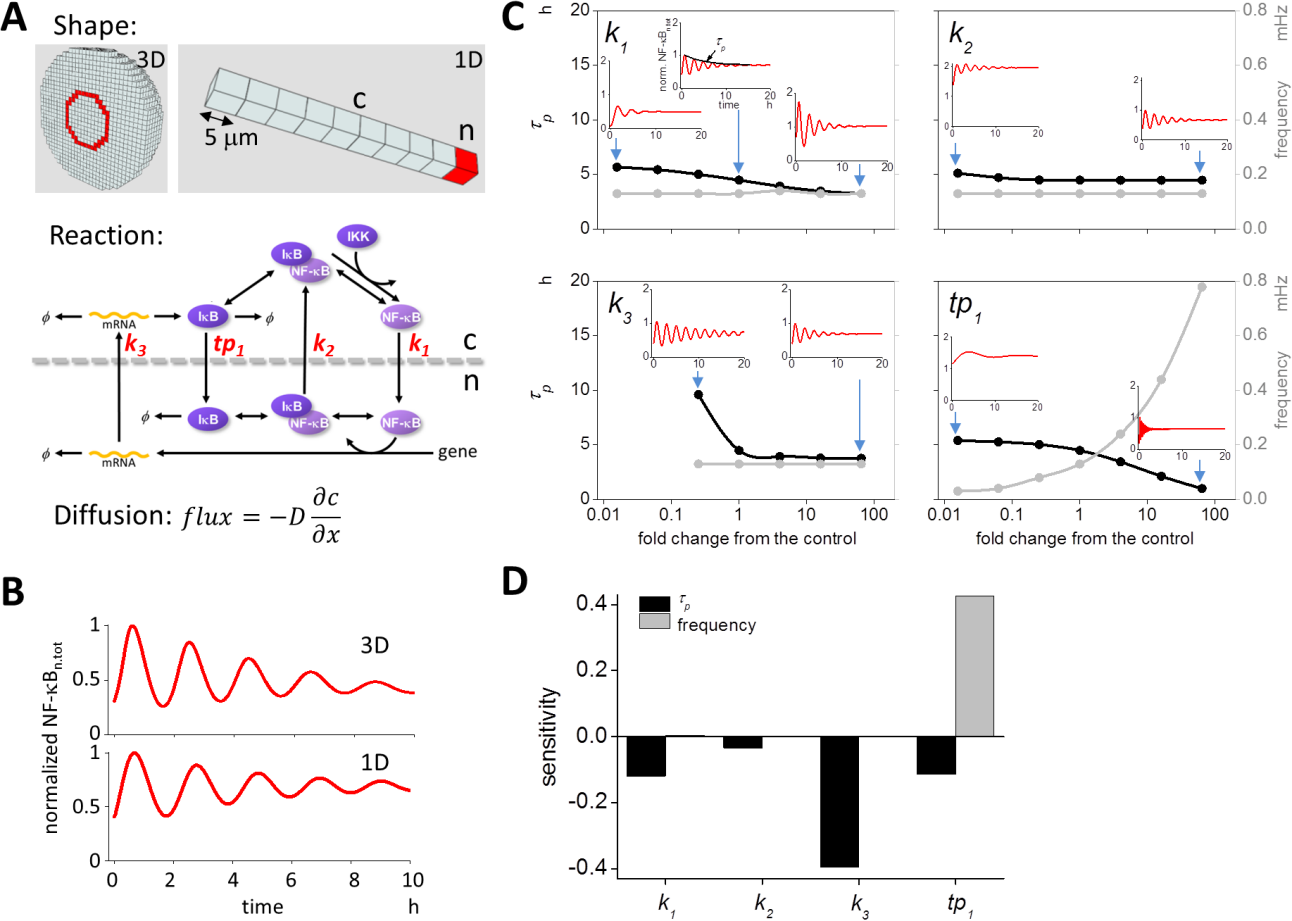
Effects of nuclear transport on the oscillation pattern of NF-κB. (A) 3D and 1D spatio-temporal models of NF-κB oscillation. The 3D shape was identical to the one used in a previous report (top left panel, [22,23]). A spherical model cell was divided into compartments enabling reaction-diffusion computational simulation. Red compartments indicates the nuclear membrane. A simplified 1D shape was employed for detail analyses (top right panel). There was a single nuclear membrane and nuclear compartment (indicated in red). Reaction schemes shown in the middle panel are identical to those presented in a previous report [23]. The effects of four kinetic rate constants (shown in red in the middle panel) were investigated. Diffusion was calculated using Fick’s equation. *D* and ∂c / ∂x represent the diffusion coefficient and spatial derivative of the concentration, respectively. (B) Simulated oscillation pattern of NF-κB_n.tot_. We obtained the same oscillation pattern from both 3D and 1D models similar to prior analyses. All parameters for the simulation were the same as previously described [23]. (C) Parameter values were changed within a range of 1/64-fold decrease to 64-fold increase of control (×1) to investigate the subsequent modification in the oscillation pattern, as characterized by *τ*_*p*_ (a measure of persistency; black lines) and frequency (gray lines). Insets show oscillations at parameter values indicated by blue arrows. The rate constants *k*_*1*_ (NF-κB import to the nucleus), *k*_*2*_ (NF-κB export from the nucleus), *k*_*3*_ (export of mRNA_IκB_ from the nucleus), and *tp*_*1*_ (the import of newly synthesized IκB to the nucleus) were investigated. (D) Sensitivity analyses of nuclear transport. Sensitivities over the entire range of each parameter were averaged. It is clear that *k*_*3*_ affected *τ*_*p*_, and *tp*_*1*_ affected the frequency.

To examine the effect of the individual components in the nuclear transport, we employed a simple spatio-temporal model of NF-κB oscillation designed to elucidate the underlying mechanisms more clearly. For this purpose, the 3D model used originally was reduced to a 1D model, as previously described (top panels of Fig 1A [23]), which included a nuclear and a nuclear membrane compartment as shown in red. Reaction schemes and rate constants were identical to those preciously shown (bottom panel of Fig 1A and S1 Fig [22,23]). We referred to the rate constants of the four pathways of nuclear transport as *k*_*1*_ (NF-κB import to the nucleus), *k*_*2*_ (NF-κB export from the nucleus), *k*_*3*_ (mRNA_IκB_ export from the nucleus), and *tp*_*1*_ (IκB import to the nucleus). We examined the changes in the oscillation pattern induced by individual changes in these rate constants. Diffusion was simulated by Fick’s equation as shown in our previous report (Fig 1A).

First, we confirmed the oscillation of NF-κB_n.tot_, which was the combined concentration of NF-κB_n_ and the IκB_n_:NF-κB_n_ complex as a measure of the fluorescence intensity of NF-κB in the nucleus. By using the previously set 1D parameters [23], we obtained oscillation of NF-κB_n.tot_ similar to those obtained in the 3D model (Fig 1B).

Next, we examined the change in the oscillation pattern of NF-κB_n.tot_ in the 1D model induced by changing the values of *k*_*1*_, *k*_*2*_, *k*_*3*_, and *tp*_*1*_. Decay time constant *τ*_*p*_ for the envelope of the peaks of oscillation waveform of NF-κB_n.tot_, which was a measure of the persistency of the oscillation, and the frequency were employed as parameters characterizing the oscillation pattern. Changing *k*_*1*_ values within a range from 1/64-fold to 64-fold of control slightly decreased *τ*_*p*_ (black line in the top left panel of Fig 1C). There was virtually no change in the frequency (gray line). Insets indicate the oscillation of NF-κB_n.tot_ at values of *k*_*1*_ indicated by blue arrows. Changing *k*_*2*_ caused almost no change in *τ*_*p*_ and frequency (top right panel of Fig 1C). Thus, the import and export of NF-κB to and from the nucleus had little or no effect on the persistency and frequency of NF-κB oscillation. In contrast, modification of *k*_*3*_ or *tp*_*1*_ resulted in a large change in the persistency and/or frequency of oscillation. Persistency was increased by a reduction in *k*_*3*_ without any appreciable change in the frequency (bottom left panel of Fig 1C). Changing the value of *tp*_*1*_ significantly modified the frequency, and this was accompanied by a change in persistency (bottom right panel of Fig 1C). Increase in *tp*_*1*_ dramatically increased the frequency of oscillation. Our 3D model provides almost identical results (S2 Fig).

These changes are clearly demonstrated by the sensitivity analysis (Fig 1D). We averaged sensitivities obtained over the entire range of parameter values. It was clear that *k*_*3*_ and *tp*_*1*_ independently regulated persistency and frequency, while *k*_*1*_ and *k*_*2*_ had only a marginal effect on these characterizing parameters. Therefore, we focused on *k*_*3*_ and *tp*_*1*_ in the following analyses.

### Changing *k*_*3*_ alters oscillation from dampened to inflating mode

**Fig 2:**
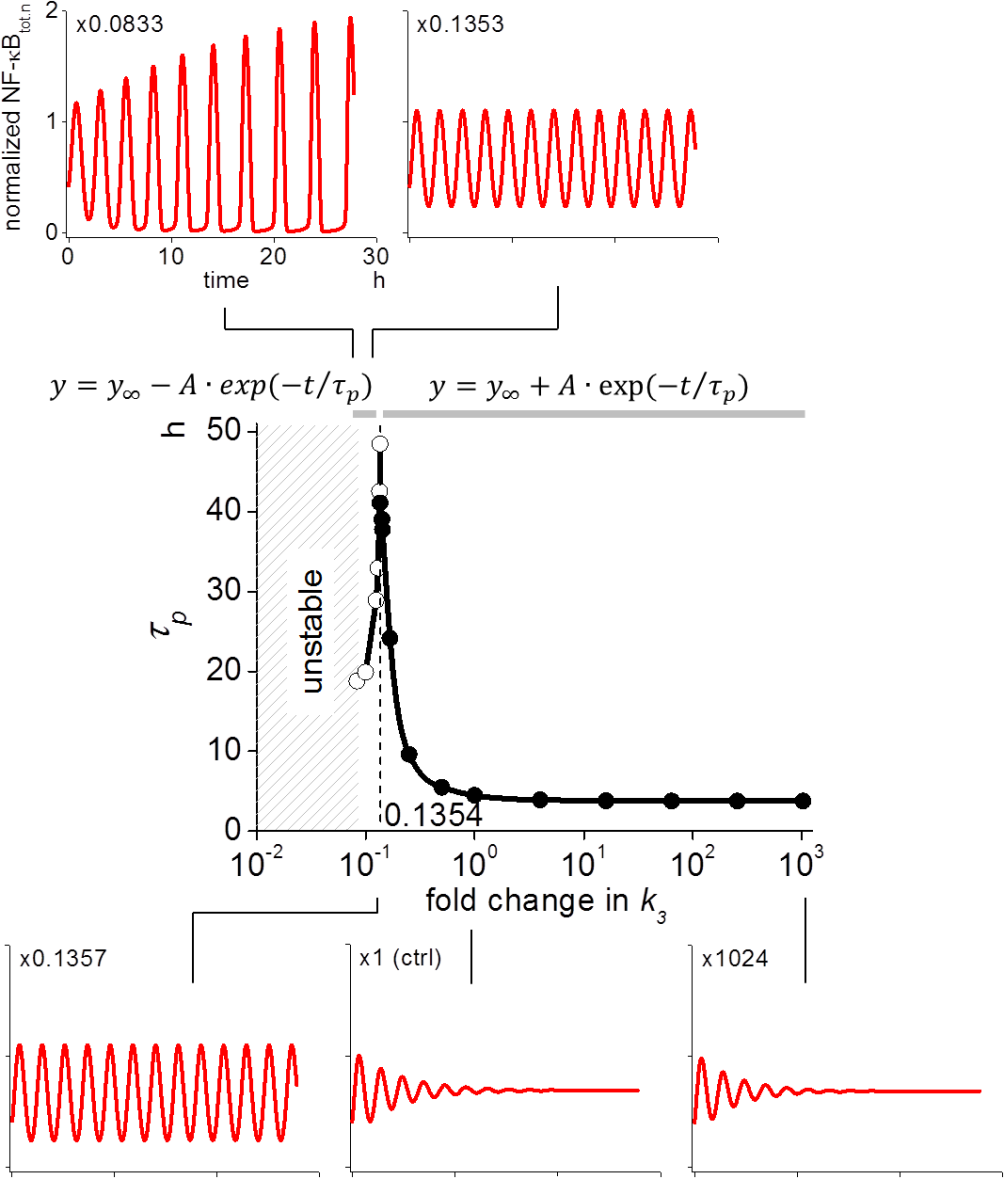
Decrease in *k*_*3*_ results in a change in the oscillation from dampened to inflating mode. Reduction of *k*_*3*_ to 0.0833-fold of the control changed the oscillation from a dampened to an inflating mode (top left panel). Decrease in *k*_*3*_ increased *τ*_*p*_ to a virtually persistent oscillation at *k*_*3*_ ~0.1354-fold of control (bottom panels and black circles in the middle panel) while increases in *k*_*3*_ from 0.0833-fold of the control resulted in the increase of *τ*_*p*_ (white circles in the middle panel). Equations shown at the top of the middle panel were used for approximating *τ*_*p*_ for inflating (left equation) and dampened (right one) oscillation by fitting the envelope of the peaks to these equations.

We found that modifying *k*_*3*_ changed *τ*_*p*_, but the change was limited to a certain range of *k*_*3*_ (Fig 1C). To investigate this further, we ran simulations at wider range of *k*_*3*_ (from 10^−2^ to 10^3^-fold change compared to the control as shown in Fig 2). We found that *τ*_*p*_ gradually increased with further decrease in *k*_*3*_. At *k*_*3*_ of 0.1357-fold of the control, the oscillation was still dampened, but seemed likely to persist for a considerably long time (bottom left panel in Fig 2). Further decrease in *k*_*3*_ resulted in a greater increase in *τ*_*p*_, which is shown by black circles in the middle panel of Fig 2. If we reduced *k*_*3*_ to 0.0833-fold of the control, however, the mode of the oscillation changed from dampened to inflating (top left panel of Fig 2). If we increased *k*_*3*_ from this point, *τ*_*p*_ was increased, shown by white circles in the middle panel of Fig 2. Thus, there was a dramatic change in the mode from dampened to inflating oscillation at *k*_*3*_ around 0.1354-fold of the control. At a *k*_*3*_ value smaller than 0.0833-fold of the control, we could not obtain a stable initial condition; that is the oscillation of NF-κB_n_ never stopped during simulations aimed at generating stable initial conditions without an NF-κB-activating stimulus.

These results were unexpected, because we simply expected that any change in *k*_*3*_ would result in a monotonic change in the persistency. It was also a surprise to find that a smaller *k*_*3*_ resulted in persistent oscillation, because smaller *k*_*3*_ was expected to result in less de novo synthesis of IκB, leading to an incomplete “reset” of NF-κB_n_ ultimately resulting in a dampened oscillation [23]. However, our simulation showed the opposite results. Therefore, we tried to elucidate this mechanism further.

### Persistent and inflating oscillation is caused by an accumulation of mRNA_IκB_ in the nucleus

To elucidate the reason for the change in the oscillation mode at smaller *k*_*3*_, we first investigated NF-κB_n_ concentration at the nucleus (c0 in S3 Fig) to see whether “reset” of NF-κB_n_ was observed during sustained oscillation as previously reported [23]. There was sufficient “reset” in the case of sustained oscillation, because the amount of NF-κB_n_ at the troughs was smaller than the initial value during sustained oscillation (gray line in the middle panel of S3 Fig), while it was higher than the initial value under conditions of dampened oscillation (black line). These results were similar to those observed following a change in the diffusion coefficient of IκB in our previous report [23]. Next we investigated the IκB concentration at the most distant cytoplasmic compartment from the nucleus (c9 in S3 Fig) to see whether the mechanism for the sustained oscillation was identical to that previously reported for a larger diffusion coefficient [23]. We found that there was no appreciable change in the IκB concentration at c9. This result differed greatly from that observed in the diffusion coefficient experiment, where a considerable amount of IκB was stored at c9 in response to a large flux of IκB due to a large diffusion coefficient, and c9 acted as a “reservoir” for IκB [23]. Thus the mechanism for sustained and/or inflating oscillation of NF-κB_n_ at a small *k*_*3*_ value was different from that for the diffusion coefficient.

**Fig 3:**
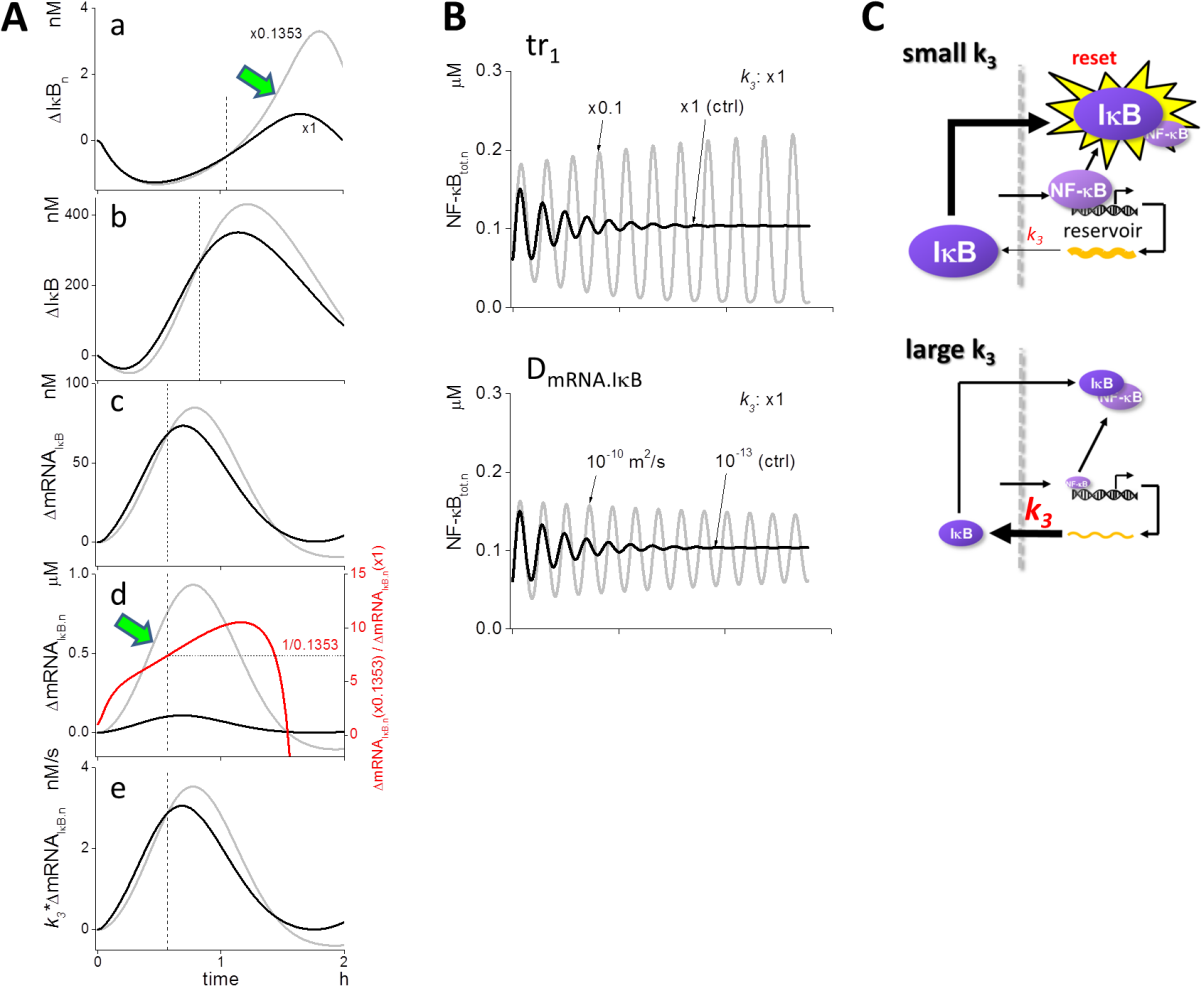
A mechanism that changes the persistency of NF-κB_n_ oscillation. (A) Time courses of concentration change relative to the initial value, for control (black lines) and 0.1353-fold decrease in *k*_*3*_ (gray lines). ΔIκB_n_ increased steeply after the crossover between *k*_*3*_ at the control and 0.1353-hold decrease (green arrow in panel a). Crossover time point is indicated by a dashed line. ΔIκB was smaller under the condition of 0.1353-fold decreased *k*_*3*_ compared to control and became larger at an earlier time point than ΔIκB_n_ (panel b). Reduced *k*_*3*_ resulted in ΔmRNA_IκB_ levels greater than control at a much earlier time point (panel c). The level of ΔmRNA_IκB.n_ was larger in decreased *k*_*3*_ from the start of oscillation and displayed a steep increase (green arrow in panel d). If we calculate the ratio of mRNA_IκB.n_ level at 0.1353-fold *k*_*3*_ to the control (red line), it reached 1/0.1353 at the time point of the crossover for mRNA (panel d). The mRNA_IκB_ flux out of the nucleus (= *k*_3_*ΔmRNA_IκB.n_) crossed-over at the same time point for both (panel e). (B) Dampened oscillation under control conditions was rescued and converted to sustained oscillation by a reduction or increase in the translation rate of IκB (*tr*_*1*_) or *D*_*mRNAIκB*_. (C) Model for the sustained oscillation driven by a small *k*_*3*_. The mRNA_IκB_ flux out of the nucleus is reduced by a small amount at reduced *k*_*3*_, allowing NF-κB_n_ to sustain mRNA_IκB_ transcription. mRNA_IκBn_ was stored in the nucleoplasmic space due to a small *k*_*3*_. Thus nucleoplasmic space acted as a “reservoir” for mRNA_IκBn_. Subsequently, accumulated mRNA_IκBn_ were exported to the cytoplasm, where large amount of IκB was newly synthesized leading to the “reset” of NF-κB_n_. This resulted in the sustained oscillation of NF-κB_n_.

To explore the specific mechanism responsible for the effects of *k*_*3*_, we investigated time courses of related species. As the “reset” of NF-κB_n_ is also important for the oscillation (middle panel of S3 Fig) as demonstrated by diffusion coefficient studies, we first carefully investigated nuclear IκB (IκB_n_), because sufficient IκB_n_ led to the “reset” of NF-κB_n_ [23]. If we compared the increase in IκB_n_ from its initial level (ΔIκB_n_ in panel a of Fig 3A), there was virtually no difference in ΔIκB_n_ between 0.1353-fold *k*_*3*_ (gray line) and the control (black line) at the beginning of the oscillation. However, it increased steeply at 0.1353-fold *k*_*3*_ (green arrow) to a value greater than control. The dashed vertical line indicates the time at which the gray and black lines crossed-over. It seemed that the delayed steep increase in ΔIκB_n_ at 0.1353-fold *k*_*3*_ was the reason for the sustained oscillation.

We investigated the time courses of the upstream species next. The increase in cytoplasmic IκB from its initial level (ΔIκB) also resembled the delayed increase at 0.1353-fold *k*_*3*_, when compared to control (panel b of Fig 3A). The same was also observed for cytoplasmic ΔmRNA_IκB_ (panel c of Fig 3A). The crossover points shifted to earlier time points as we investigated the upstream species. If we compared these with the nuclear ΔmRNA_IκB.n_, however, the increase was much more steep at 0.1353-fold *k*_*3*_ (gray line and green arrow in panel d of Fig 3A) than in control (black line), and there was no crossover at a time earlier than that observed for cytoplasmic ΔmRNA_IκB_. It is important to note that when we calculated the ratio of the increase in ΔmRNA_IκB.n_ for both the cases, it was 1/0.1353 at the same time point as the crossover observed in conjunction with the increase of cytoplasmic ΔmRNA_IκB_ (red curve and dashed line). We then calculated the flux of ΔmRNA_IκB_ from the nucleus by multiplying *k*_*3*_ with ΔmNRA_IκB.n_ (panel e of Fig 3A). Fluxes at 0.1353-fold *k*_*3*_ and the control crossed over at the same time point as the crossover observed when the ratio of the increase in ΔmRNA_IκB.n_ was 1/0.1353. Thus, the delayed increase in IκB (panel b of Fig 3A) was caused by the sustained transcription of mRNA_IκB_ (panel d of Fig 3A), which was caused by the small *k*_*3*_.

We were surprised to find that a smaller *k*_*3*_ value resulted in the sustained oscillation. However, the mechanism thus revealed was reasonable to account for the change in the oscillation mode by *k*_*3*_. If this proposed mechanism holds true, a lower rate of de novo synthesis of IκB would also result in sustained transcription of mRNA_IκB_ leading to the sustained oscillation of NF-κB_n_. This was true when *tr*_*1*_, which was the translation rate of IκB (S1 Fig), was decreased to one-tenth to control; dampened oscillation at control was rescued to sustained and inflating oscillation (top panel of Fig 3B). If *D*_*mRNA*.*IκB*_, the diffusion coefficient of IκB mRNA, was larger, mRNA_IκB_ would diffuse to distant locations within the cytoplasm, and the de novo synthesis of IκB should be reduced, leading to the sustained transcription of mRNA_IκB_ and a sustained oscillation. This hypothesis also held true, as shown in the bottom panel of Fig 3B. In both the cases, we observed delayed increases in IκB_n_ and mRNA_IκB.n_, as in the case of decreased *tr*_*1*_, and NF-κB_n_ level at troughs was lower than that of the control (S4 Fig). Thus, all three of the parameters tested, *k*_*3*_, *tr*_*1*_, and *D*_*mRNA*.*IκB*_ contributed to the sustained oscillation by the same mechanism.

In summary, when *k*_*3*_ was small, the mRNA_IκB_ flux out of the nucleus was decreased leading to less de novo synthesis of IκB and a sustained transcription of mRNA_IκB_ that was stored in the nucleus. This then led to the “reset” of NF-κB by an increase in de novo synthesis of IκB at subsequent time points, as well as to the sustained oscillation of NF-κB. Thus, a small *k*_*3*_ resulted in the use of nucleoplasmic space as a “reservoir” for mRNA_IκB_ (Fig 3C). Larger *k*_*3*_ enabled greater mRNA_IκB_ flux out of the nucleus, and early synthesis of IκB. This then prohibited sustained transcription of mRNA_IκB_ by NF-κB_n_, leading to the dampened oscillation.

Finally, we compared the persistency of the observed oscillation following changes in *k*_*1*_, *k*_*2*_, and *tp*_*1*_, since these parameters did not appear to have a major effect on persistency (Fig 1C). As shown in S5 Fig, neither a “reset” of NF-κB_n_ nor a steep increase in mRNA_IκB.n_ was observed after the start of the oscillation. In particular, no change in the time course of NF-κB_n_ and mRNA_IκB.n_ was induced following a change in *k*_*2*_. Thus, the change in the persistency was specific to the nuclear transport induced by *k*_*3*_ in our model.

### Modification of *tp*_*1*_ causes a large change in the oscillation frequency

**Fig 4:**
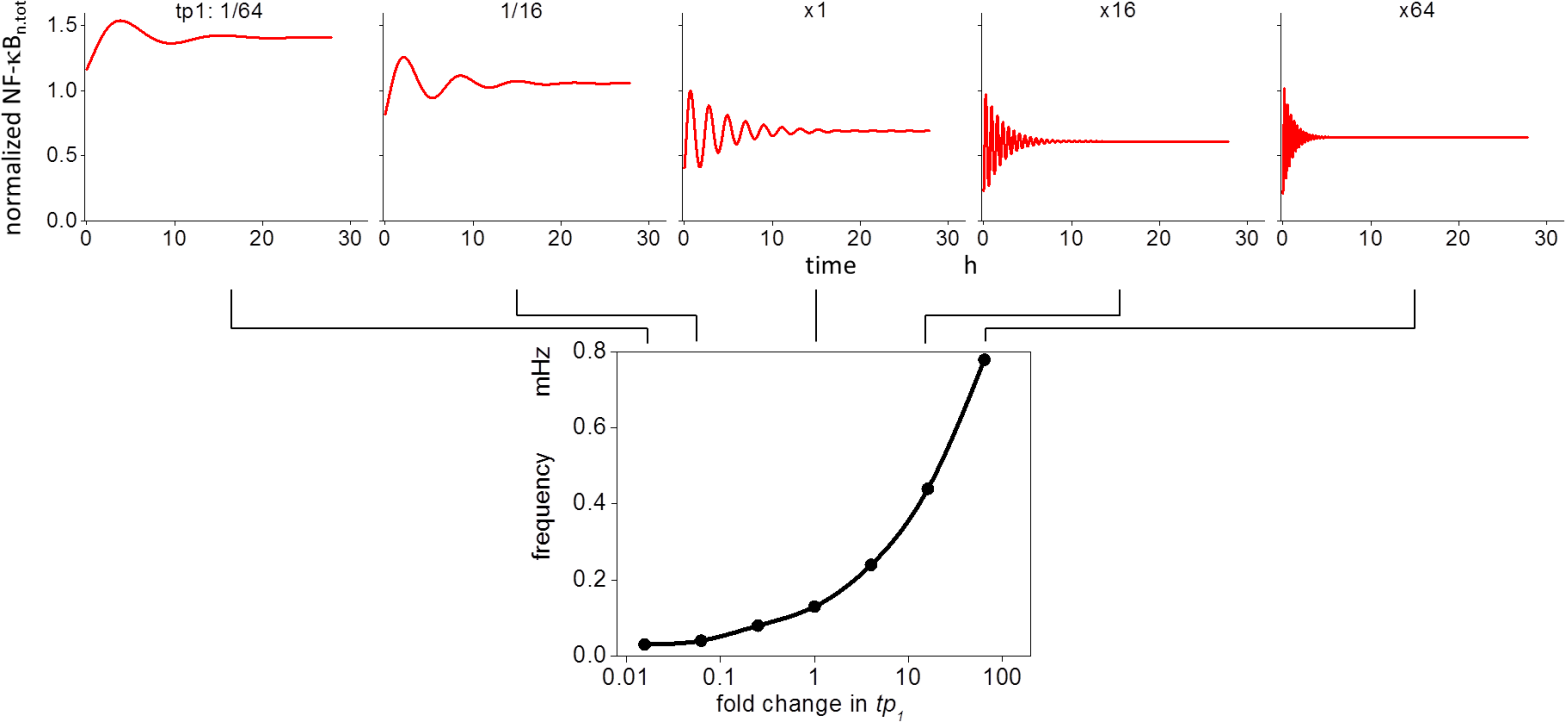
Modification of *tp*_*1*_ changes the oscillation frequency of NF-κB_n_. Increasing *tp*_*1*_ increased the oscillation frequency, while the average level of NF-κB_n_ was decreased at the same time. A greater than 20-fold change in the frequency was observed.

Our earlier results demonstrated that changes in *tp*_*1*_ affected the oscillation frequency (Fig 1C). To investigate the effect further, we modified the *tp*_*1*_ values, and the changes in the oscillation patterns for various *tp*_*1*_ values are shown in Fig 4. Oscillation frequency increased with an increase in *tp*_*1*_, while the average level of NF-κB_n.tot_ was decreased. A greater than 20-fold increase in the frequency resulted from a change in *tp*_*1*_, ranging from a 1/64-fold decrease to a 64-fold increase from the control. We then explored the reason for the change in frequency by *tp*_*1*_.

### Increase in the change in slope of NF-κB_n_ contributes to increased oscillation frequency

**Fig 5:**
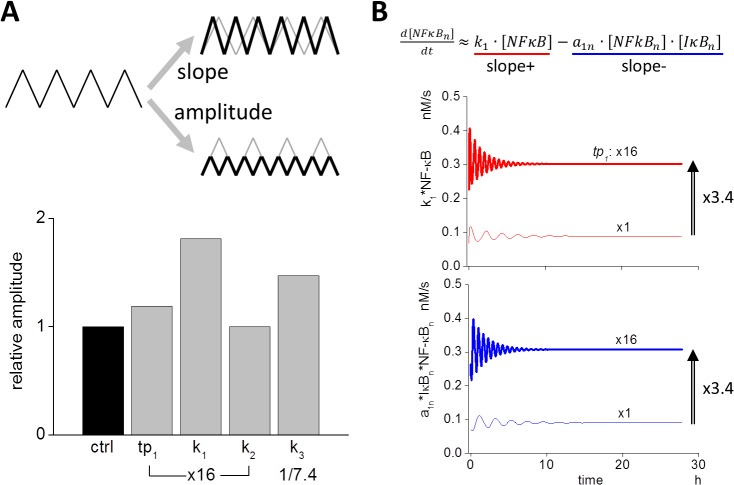
Slopes and amplitude of oscillation waveform of NF-κB_n_ determine frequency. (A) If increasing (slope+) and/or decreasing slopes (slope-) are steeper without a corresponding change in amplitude, oscillation frequency increases. Conversely, if the amplitude decreases without changing slopes, the frequency also increases (upper panel). The change in amplitude resulting from the modifications in each of the four parameters are shown (bottom panel). While a 16-fold increase in *tp*_*1*_ resulted in higher frequency, the change in the amplitude was small. While there was virtually no change in the frequency in response to modifications of *k*_*1*_ and *k*_*3*_, an increase in *k*_*1*_ or a decrease in *k*_*3*_ resulted in the increase in the amplitude. (B) Slope+ and slope- were approximated by the inward flux of *k*_1_*NF-κB and the flux of complex formation *a*_1n_*NF-κB_n_*IκB_n_ (see main text). When *tp*_*1*_ was increased 16-fold, both inward and outward fluxes were increased (lower two panels).

There are two ways to change the frequency of NF-κB_n_ oscillation, namely, the slope and the amplitude (upper panel of Fig 5A). If the slope increases without a change in amplitude, oscillation frequency increases. If the amplitude decreases without a change in the slope, the oscillation frequency increases as well. Therefore, we first compared the amplitude that resulted from a change in the four parameters *tp*_*1*_, *k*_*1*_, *k*_*2*_, and *k*_*3*_ (lower panel of Fig 5 and S6 Fig). While an increase in amplitude was observed following an increase in *k*_*1*_ or a decrease in *k*_*3*_, respectively, virtually no change in amplitude was observed following a change in *tp*_*1*_ and *k*_*2*_. Therefore, a reduction in the amplitude caused by a change in *tp*_*1*_ was not the reason for the increased oscillation frequency.

Next we investigated the slope of NF-κB_n_. Rising (slope+) and falling (slope-) slopes were analyzed independently (S7A Fig). Slope+ and slope- were direct consequences of the inward and outward fluxes of NF-κB and IκB_n_:NF-κB_n_, respectively, which were calculated by *k*_1_*NF-κB and *k*_2_*IκB_n_:NF-κB_n_. If we compared NF-κB_n.tot_, which was the summation of IκB_n_:NF-κB_n_ and NF-κB_n_ with NF-κB_n_ alone, there was only a small difference between the two (S7B Fig). In addition, *d*_1n_*NF-κB_n_:IκB_n_ was negligible in comparison to *a*_1n_*NF-κB_n_*IκB_n_ (**Materials and Methods**). We then approximated the change in NF-κB_n.tot_ by using NF-κB_n_, which was further approximated by Eq 1 (**Materials and Methods**). Thus we employed *k*_1_*NF-κB and *a*_1n_*NF-κB_n_*IκB_n_ as slope+ and slope-, respectively (Fig 5B).

**Fig 6:**
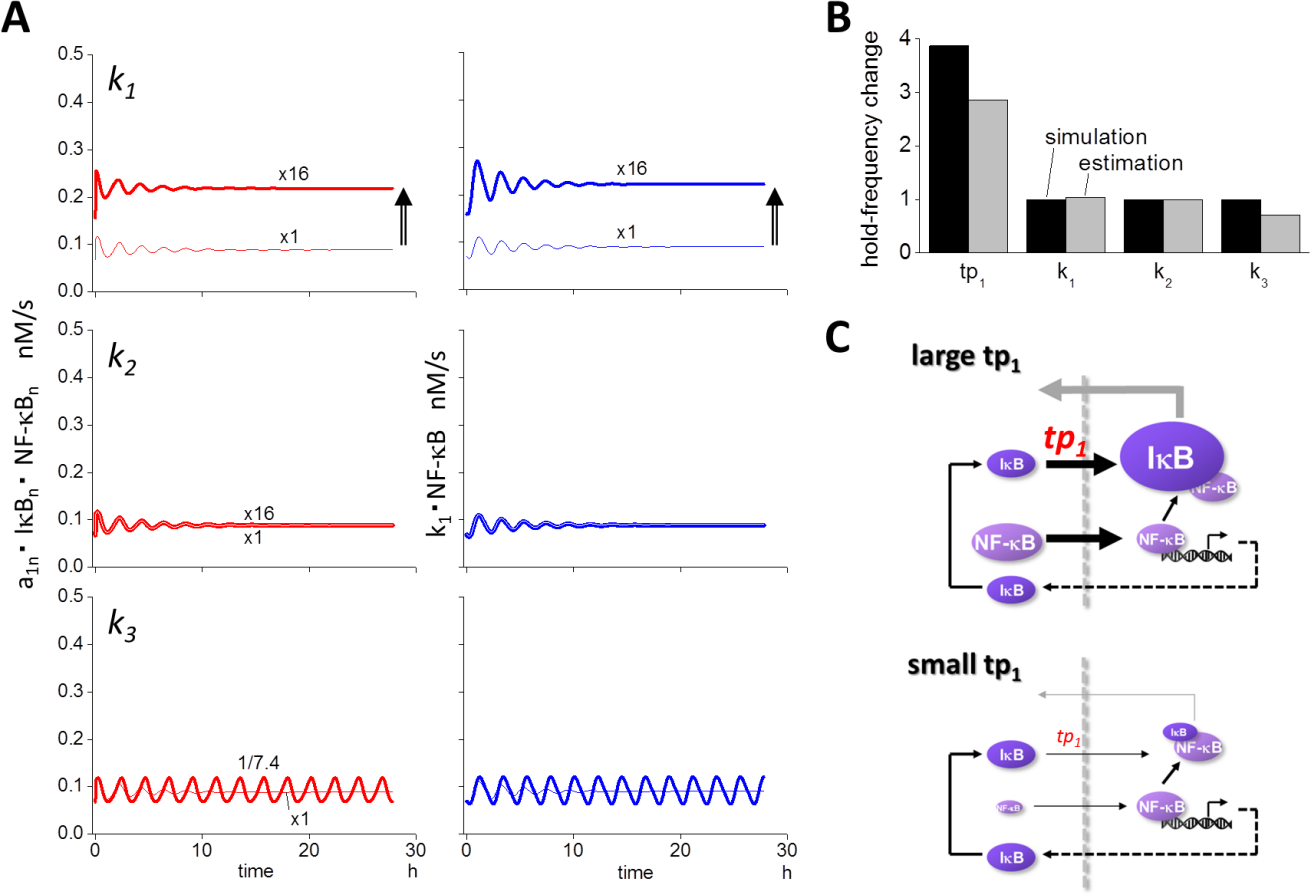
Estimated changes in the frequency resulting from changes in slopes and amplitude agree with simulations. (A) Slope+ and slope- were larger given a 16-fold increase in *k*_*1*_, while there was virtually no change in the slopes following a 16-fold increase or a 1/7.4-fold decrease in *k*_*2*_ or *k*_*3*_. (B) The change in the oscillation frequency was estimated by the change in slopes and amplitude (see main text). There was a reasonable agreement between simulation (black bars) and estimation (gray bars). (C) Summary of the mechanism of changes in the oscillation frequency generated by modifications of *tp*_*1*_. Increasing *tp*_*1*_ relocated NF-κB to the cytoplasm resulting in an increase in its inward flux to the nucleus. In addition, a larger *tp*_*1*_ value directly resulted in an increase in the inward flux of IκB to the nucleus, resulting in a larger amount of IκB_n_ and leading to an increase in the outward flux of NF-κB_n.tot_.

Following the 16-fold increase in *tp*_*1*_, an increase of 3.40-fold was observed both in *k*_1_*NF-κB and *a*_1n_*NF-κB_n_*IκB_n_, indicating that both slope+ and slope- became steeper with the same magnitude (Fig 5B). If we compared slope+ and slope- for the 16-fold increase in *k*_*1*_, increases to the same degree were observed for both slopes. However, no change in these slopes were obtained from the 16-fold increase in *k*_*2*_ and the 1/7.4-fold decrease in *k*_*3*_ (Fig 6A). These are summarized in S8 Fig, together with the change in the slopes as designated by *tp*_*1*_.

Since we hypothesized that the oscillation frequency could be calculated by using slopes and amplitude by a simplified oscillating waveform with triangle wave, we estimated oscillation frequency by Eq 2 (**Materials and Methods**, S9 Fig). Estimated frequencies for *tp*_*1*_, *k*_*1*_, *k*_*2*_, and *k*_*3*_ agreed reasonably with frequencies obtained by simulations (Fig 6B). With a large *k*_*1*_, both the amplitude and the slopes were increased to almost the same extent (S9 Fig) and no change in the frequency was observed. Modification of *tp*_*1*_, however, resulted in an appreciable change only in the slopes but not in the amplitude. Therefore, it is clear that the frequency was altered by a change in *tp*_*1*_.

Under conditions of high *tp*_*1*_ and *k*_*1*_, NF-κB flux into the nucleus was increased (Figs 5B and 6A). Increase in the flux was obviously caused by the increase in *k*_*1*_. However, why did high *tp*_*1*_ also increase flux? As shown in S10 Fig, larger *tp*_*1*_ caused greater IκB flux into the nucleus leading to an increase in IκB_n_. This then led to increased IκB_n_:NF-κB_n_ flux out of the nucleus which subsequently reduced NF-κB_n_ and increased NF-κB in the cytoplasm. Thus, the preferred storage site of NF-κB was relocated to the cytoplasm. In summary, slope+ and slope- were increased by an increase in cytoplasmic NF-κB and increase in the IκB flux into the nucleus, which was the direct consequence of the increase in *k*_*1*_ and *tp*_*1*_, respectively. This was the major mechanism that drove higher oscillation frequency of NF-κB_n.tot_ (Fig 6C). Under conditions of a smaller *tp*_*1*_, both the average level of cytoplasmic NF-κB and the inward flux of IκB toward the nucleus were small resulting in a lower frequency. Thus, *tp*_*1*_ regulated the oscillation frequency by two different mechanisms for slope+ and slope-.

## Discussion

**Fig 7:**
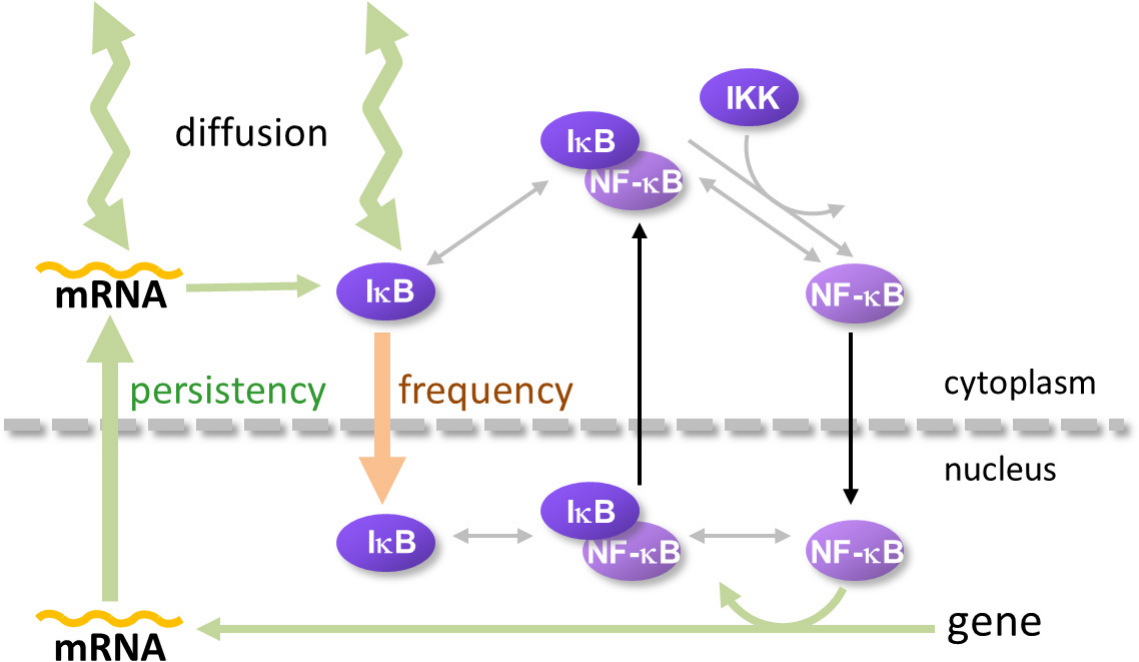
Mechanisms regulating the oscillation pattern of nuclear NF-κB. The present study together with our previous one [23], showed distinct spatial parameters regulating the persistency and frequency of NF-κB_n_ oscillation. *D*_*mRNAIκB*_ and *D*_*IκB*_ (thick zigzag light green arrows) and the rate of mRNA_IκB_ efflux (thick light green arrow) are spatial parameters that regulate the persistency of the oscillation. The rate of the influx of IκB (light brown arrow) is a spatial parameter that regulates frequency. In addition, transcription of mRNA_IκB_ and translation of IκB are non-spatial parameters regulating persistency (thin light green arrows). There was virtually no effect on persistency and the frequency by other nuclear membrane transport mechanisms (black arrows).

We have been investigating the mechanisms that change the oscillation patterns of NF-κB following modification of spatial parameters ([22,23] and this report). Over the course of these analyses we found that the “reset” of NF-κB_n_ was important for the sustained oscillation, and larger *D*_*IκB*_ helped to “reset” NF-κB_n_ by storing newly synthesized IκB at a cytoplasmic space distant from the nucleus, which acted as a “reservoir” [23]. Here we report that the efflux of mRNA_IκB_ and influx of IκB from and to the nucleus independently regulate the persistency and frequency of oscillation (Fig 1C and 1D). In addition, the reduction in the rate of translation and increase in *D*_*mRNA*.*IκB*_ increase the persistency of oscillation. Increase in *D*_*IκB*_ rescued the heavily-dampened oscillation as shown in the previous report [23]. Using these analyses we developed a model to explain the regulation of the oscillation pattern by spatial parameters, as summarized in Fig 7. The model indicates that distinct spatial parameters regulate the persistency and frequency of NF-κB_n_ oscillation. *D*_*mRNA*.*IκB*_ and *D*_*IκB*_ and the rate of mRNA_IκB_ efflux are spatial parameters that regulate the persistency, which are shown in green arrows, and the rate of the influx of IκB is a spatial parameter that regulates the frequency of the oscillation, which is shown in a brown arrow. Transcription of mRNA_IκB_ and the translation of IκB are non-spatial parameters regulating the persistency of oscillation. There is virtually no effect by any other nuclear membrane transport mechanisms on the persistency and the frequency, which is shown in black arrows. In summary, our results suggest that the mechanisms regulating IκB are responsible for the regulation of the oscillation pattern.

In our studies, the rate of transcription was calculated by the equation shown at the top of S11 Fig, which was identical to that used in a previous report [22,23]. If we changed the “n” from the control value of 2 to 1 or 3 at the 0.1353-fold reduced *k*_*3*_ condition, the sustained oscillation was modified to a dampened or an inflating oscillation, respectively (bottom panels in S11 Fig). Thus “n", the parameter describing how many NF-κB molecules bound to the κB site of DNA were required for transcription, seems to regulate persistency.

We observed an inflating oscillation of NF-κB in the 1D model following a low flux of mRNA_IκB_ nuclear export (Fig 2). The same inflating oscillation was observed in our 3D model when IκB kinase (IKK) degradation was ignored (S12 Fig). Thus, the inflating oscillation was not unique to the 1D model, but could also be observed in the 3D model provided that the effects of IKK degradation were negligible.

Together with our previous results [23], our current study demonstrates that the frequency and persistency of NF-κB oscillation can be regulated by its inhibitor molecule IκB and mRNA_IκB_. There was only a marginal effect by parameters directly regulating NF-κB, such as its rate of influx and efflux. These results might seem to be counterintuitive, as rates of the influx and efflux of NF-κB were expected to directly regulate NF-κB oscillation. However, our analyses revealed that IκB predominantly regulated the oscillation pattern. This further suggests that currently unknown mechanisms regulating the concentration and/or dynamics of IκB might also regulate frequency and/or persistency.

In the present study, we have analyzed effects of fluxes through the nuclear envelope (NE) by changing the corresponding rate constants (Fig 1C). There are two ways to alter the flux through NE: by changing the density/number of NPCs or by altering the rate of the flux through a single NPC. Senescence has been shown to decrease the number of NPCs [26], which suggests that it could decrease the outward flux of IκB mRNA and inward flux of IκB protein, together with other proteins and mRNAs. Since the inward and outward fluxes of NF-κB have virtually no effect on the oscillation pattern (Fig 1C), senescence could potentially lead to a persistent and low-frequency oscillation of NF-κB. A previous study has shown that the leukemogenic Nup98 fusion proteins caused an aberrant localization of the CRM1 protein [27]. Although the mechanism of nuclear export of IκB mRNA is largely unknown, CRM1 and NXF1 are possibly involved in this process, as shown in other mRNA export studies [29,30], and the export of IκB mRNA could be retarded in cells expressing leukemogenic Nup98 fusion proteins leading to the persistent oscillation of NF-κB. Thus, the nuclear transport could play an important role in some diseases and in aging of the cell.

It is important to address possible experimental procedures to alter the nuclear transport of IκB mRNA and IκB protein selectively. Although mechanisms of nuclear import of IκB are not well known [31], it was reported that the nuclear import of IκBα is temperature and ATP dependent and is blocked by a dominant-negative mutant of importin β [32]. Thus, the efficacy of IκBα import could be regulated by the expression level of a dominant-negative mutant of importin β. The export of mRNA and protein complex (mRNP) is not simple, but is composed of many steps, including the proper assembly of a mRNP, and its targeting and docking to NPC [29,30]. If the binding of CRM1 or NXF1 to IκB mRNP is blocked partially, its export could be selectively impaired leading to the persistent oscillation of NF-κB. In fact, it is reported that the export of mRNPs is influenced by elements within the mRNA, which is responsible for the binding of CRM1 or NXF1 to mRNA [29]. If the element in IκB mRNA responsible for this process is mutated, its export could be retarded leading to the persistent oscillation of NF-κB. The diffusion coefficient is inherent to the molecular species. However, its effective value can be modified by a change in the effective size. If IκB mRNP is enlarged by the binding of non-functional proteins, the effective value of *D*_*mRNA*.*IκB*_ will be reduced because of hindered diffusion. This could lead to the dampened oscillation of NF-κB.

In conclusion, our investigations on the possible regulatory mechanisms of NF-κB_n_ oscillation revealed that the export of IκB mRNA from the nucleus and the import of IκB to the nucleus are important in regulating the persistency and frequency of the oscillation. A decrease in the export of IκB mRNA facilitated an enhanced transcription by NF-κB_n_, which was retained in the nucleus, to be subsequently exported to the cytoplasm to “reset” NF-κB_n_ and to sustain the persistency of the oscillation. Conversely, an increase in the influx of IκB led to increases in the influx and efflux of NF-κB resulting in the higher oscillation frequency. These results provide a better understanding of the mechanism of NF-κB_n_ oscillation and the importance of the nuclear transport, indicating its relevance in the understanding of disease mechanisms.

## Materials and Methods

### Computational model

We constructed spatio-temporal 3D and 1D computational models of NF-κB oscillation as previously described [23]. We used the same chemical reaction model as in the previous report (S1 Fig, [23]). Briefly, the model comprised the formation of IKK:IκBα:NF-κB complex, the degradation of IκBα and the subsequent nuclear transportation of NF-κB, NF-κB transcription of IκBα mRNA, IκBα protein synthesis, and the nuclear export of the IκBα:NF-κB complex. We employed a simplified chemical reaction model excluding A20 and CYLD as our model was intended to extract phenomena and mechanisms for the regulation of the NF-κB oscillation pattern by nuclear transport.

The 3D spherical cell model with a diameter of 50 μm was divided into small cubic compartments (total 62,417) of identical size enabling reaction-diffusion simulations (top left panel of Fig 1A). We used Fick’s equation for simulating diffusion, which was combined with differential equations for the chemical reactions. The central 8.3% of the compartments was assigned as the nucleus. In the 1D model, which was used for the analysis of the effect of nuclear transport, there were 10 cubic compartments with an edge length of 5 μm per cube, and the rightmost red compartment was assigned as the nucleus and nuclear membrane compartment (top right panel of Fig 1A). Reaction schemes shown in S1 Fig were embedded in the corresponding regions of the cytoplasm, nuclear membrane, and nucleus of the 3D and 1D models.

We employed the 1D model for the efficiency of analyses, because there were only 1/6241.7^th^ compartments in the 1D model compared to the 3D model. All models were constructed using A-Cell software [33,34]. Models and all parameters used in the present study can be downloaded from http://dx.doi.org/10.6084/m9.figshare.1417973. Kinetic parameters used in our simulation were the same as in the previous report [23].

### Simulations

Simulation programs in c language were automatically generated by A-Cell. We used the parallelized version by openMP for a multi-core CPU. Simulations were run on a Linux computer equipped with an Intel compiler. Every time we changed parameters for nuclear transport, we first acquired an equilibrium forcing IKK = 0, which ensured a resting state. Thereafter a simulation of NF-κB oscillations was run by setting concentrations acquired by equilibration. Simulated concentrations of nuclear NF-κB were plotted as values normalized to the maximum at the control condition, unless otherwise noted.

### Analyses

Frequency of NF-κB_n_ oscillation was analyzed by FFT (Fast Fourier Transform) using Origin8.5J by OriginLab Corp. The time constant of dampened or inflating oscillation was fitted to equations shown in Fig 2. Time series data of peak amplitude were extracted and fitted to either equation by using the curve-fit package of Origin 8.5J (inset in the top left panel of Fig 1C showing *τ*_*p*_).

### Calculation of slopes in the oscillation waveform and estimation of oscillation frequency

The slope of the oscillation was defined as the rate of the increase or decrease in the total concentration of nuclear NF-κB (NF-κB_n.tot_), which was the summation of NF-κB and its complex IκB:NF-κB in the nucleus. Thus, the rate change in NF-κB_n.tot_ was calculated by

$$\frac{d\left[NF\kappa B_{n.tot}\right]}{dt}=\frac{d\left(\left[I\kappa B_{n}:NF\kappa B_{n}\right]+\left[NF\kappa B_{n}\right]\right)}{dt}.$$

Subscription n indicates species in the nucleus. Since there was only a small difference between NF-κB_n.tot_ and NF-κB_n_ (S7B Fig), we reduced the equation as follows:

$$\frac{d\left[NF\kappa B_{n.tot}\right]}{dt}\approx \frac{d\left[NF\kappa B_{n}\right]}{dt}=k_{1}∙\left[NF\kappa B\right]-a_{1n}∙\left[I\kappa B_{n}\right]\left[NF\kappa B_{n}\right]+d_{1n}∙\left[I\kappa B_{n}:NF\kappa B_{n}\right].$$

If we compared the 2^nd^ and the 3^rd^ terms, the 3^rd^ term was negligibly small (9.04 × 10^−11^ v.s. 1.59 × 10^−12^ M/s). Therefore, we further simplified the equation as follows:

$$\frac{d\left[NF\kappa B_{n.tot}\right]}{dt}\approx k_{1}∙\left[NF\kappa B\right]-a_{1n}∙\left[I\kappa B_{n}\right]\left[NF\kappa B_{n}\right].$$ Eq.1)

The 1^st^ and 2^nd^ terms on the right hand side were the positive (slope+) and negative (slope-) slopes, respectively (Fig 5B).

If we simplified the oscillation waveform of NF-κB by triangular wave (top panel of Fig 5A), the change in the oscillation frequency was proportional and inversely proportional to the average slope of slope+ and slope- and amplitude. Therefore, we calculated the estimated fold change in the frequency by`

$$\text{est}.\;\text{fold change in the freq}.=\frac{fold\;change\;in\;\left\{\left(slope+\right)+\left(slope-\right)\right\}/2}{fold\;change\;in\;amplitude}.$$ Eq.2)

## Supporting Information

S1 Fig Reaction scheme for the oscillation of NF-κB_n_ in the 1D model showing rate constants and diffusion coefficients. The scheme is identical to that used in a previous report [23]. The diffusion process both for mRNA_IκB_ (t.IκB) and protein IκB are explicitly shown by zigzag lines with bidirectional arrowheads, since these played an important role in the persistency of NF-κB_n_ oscillation (see text). Other species diffused with diffusion coefficients of 10^−11^ m^2^/s.
(TIF)

S2 Fig Effects of nuclear transport on *τ*_*p*_ and frequency in the 3D model. Simulation results of *τ*_*p*_ (black asterisks) and frequency (red asterisks) at selected value of each parameter in the 3D model are shown together with those used in the 1D simulations (black and gray circles). Although there were discrepancies between 1D and 3D simulations in *k*_*3*_ at 0.25-fold decrease and *tp*_*1*_ at 16-fold increase, the overall propensity of the change agreed between the 3D and 1D simulations.
(TIF)

S3 Fig The mechanism for sustained and inflating oscillation of NF-κB differs from that of the diffusion coefficient. At c0 (nuclear compartment), the NF-κB_n_ concentration at the troughs was larger for the dampened oscillation and smaller for the sustained oscillation at control value of *k*_*3*_ and 0.1353-fold of the control, respectively (middle panel), similar to the case of diffusion coefficient. However, there was no appreciable difference in the average level of IκB at c9 (most distant cytoplasmic compartment) in both oscillations (bottom panel), which was different from the case of diffusion coefficient.
(TIF)

S4 Fig Decreasing *tr*_*1*_ or increasing *D*_*mRNA*.*IκB*_ leads to sustained oscillation by the same mechanisms observed with a change in *k*_*3*_. We found the same steep increases in IκB_n_ and mRNA_IκB.n_ (green arrows) caused by the decreasing or increasing *tr*_*1*_ (A) or *D*_*mRNA*.*IκB*_ (B) at the control level of *k*_*3*_. The levels of NF-κB_n_ were lower at troughs than initial levels indicating a sufficient “reset” under these conditions, which led to persistent oscillation.
(TIF)

S5 Fig No persistent oscillation following a change in *k*_*1*_, *k*_*2*_, or *tp*_*1*_. Neither “reset” nor a steep increase in mRNA_IkB.n_ was observed following changes in these parameters. The time course of NF-kB and mRNA_IkBn_ overlapped almost completely after a reduction of *k*_*2*_ to 1/16 (middle panels).
(TIF)

S6 Fig Change in the amplitude of NF-κB_n.tot_ oscillation generated by changes in the four parameters of nuclear membrane transport. There was virtually no change in the amplitude following changes in *tp*_*1*_ and *k*_*2*_, and there was a small change in the amplitude following changes in *k*_*1*_ and *k*_*3*_. Thin black and thick gray lines indicate the NF-κB_n.tot_ oscillation under control conditions and at the 16-fold increase (*tp*_*1*_, *k*_*1*_, and *k*_*2*_) or 1/7.4-fold decrease (*k*_*3*_).
(TIF)

S7 Fig Factors regulating the slope of NF-κB_n.tot_ oscillation. (A) If we approximated the oscillation by a triangular waveform, two slopes (slope+ and slope-) regulated the frequency. Slope+ and slope- were directly calculated by the inward and outward fluxes of NF-κB (*k*_1_*NF-κB) and IκB_n_:NF-κB_n_ complex (*k*_2_*IκB_n_:NF-κB_n_). (B) There was almost no difference between NF-κB_n.tot_ and NF-κB_n_ indicating that we could perform the analyses using NF-κB_n_ instead of NF-κB_n.tot_.
(TIF)

S8 Fig Calculation of slope+ and slope-. To generate slope+ and slope-, we needed to know the concentrations of NF-κB (top), NF-κB_n_ (middle), and IκB_n_ (bottom). We used average concentrations at equilibrium. Estimated slopes relative to the control values are listed. While a 3.40-fold increase in slope+ and slope- resulted from a 16-fold increase in *tp*_*1*_, virtually no change occurred following a 16-fold increase in *k*_*2*_ and a 0.1353-fold decrease in *k*_*3*_. Increase in *k*_*1*_ generated marginal changes.
(TIF)

S9 Fig Estimated change in the frequency. Red and blue lines are slope+ and slope- under control (thin lines) and changed conditions (thick lines) designated in each panel. Estimated changes in the frequency (est.freq.) were calculated by the change in the amplitude and the average slope by Eq 2. Only change in *tp*_*1*_ resulted in an appreciable change in the frequency.
(TIF)

S10 Fig Mechanism that relocates NF-κB after a change in *tp*_*1*_. 1) Larger *tp*_*1*_ increased the inward flux of IκB resulting in the reduction of cytoplasmic IκB. 2) This increased IκB_n_ led to the reduction of NF-κB_n_ due to the increase in the efflux of NF-κB_n_. 3) Because of the increase in the NF-κB_n_ efflux, the cytoplasmic NF-κB increased. Thus, the equilibrium changed to a state of greater cytoplasmic NF-κB.
(TIF)

S11 Fig A change in mRNA_IκB_ transcription alters the persistency of the oscillation. Transcription of mRNA_IκB_ was calculated by the equation shown above. There were two parameters controlling the transcription, *tr*_*2*_ and n. Among them, n described the nonlinearity of the transcription in relation to the concentration of NF-κB_n_. n = 2 at the control condition, assuming that the binding of two NF-κB molecules to the κB site of genes was required for their regulation. If n was set to 1 or 3, the sustained oscillation at 0.1353-fold decreased *k*_*3*_ resulted in a dampened or an inflating oscillation (left and right panels, respectively).
(TIF)

S12 Fig Inflating oscillation in the 3D model. When the rate of IκB kinase (IKK) degradation was set to 0 as in the 1D model, inflating oscillation was observed in the 3D model as well.
(TIF)
