# Supplementary figures and images for: Regulation of NF-κB Oscillation by Nuclear Transport: Mechanisms Determining the Persistency and Frequency of Oscillation
Source: PLoS One. 2015 Jun 4;10(6):e0127633. doi: 10.1371/journal.pone.0127633 (PMC4456371; doi:10.1371/journal.pone.0127633)

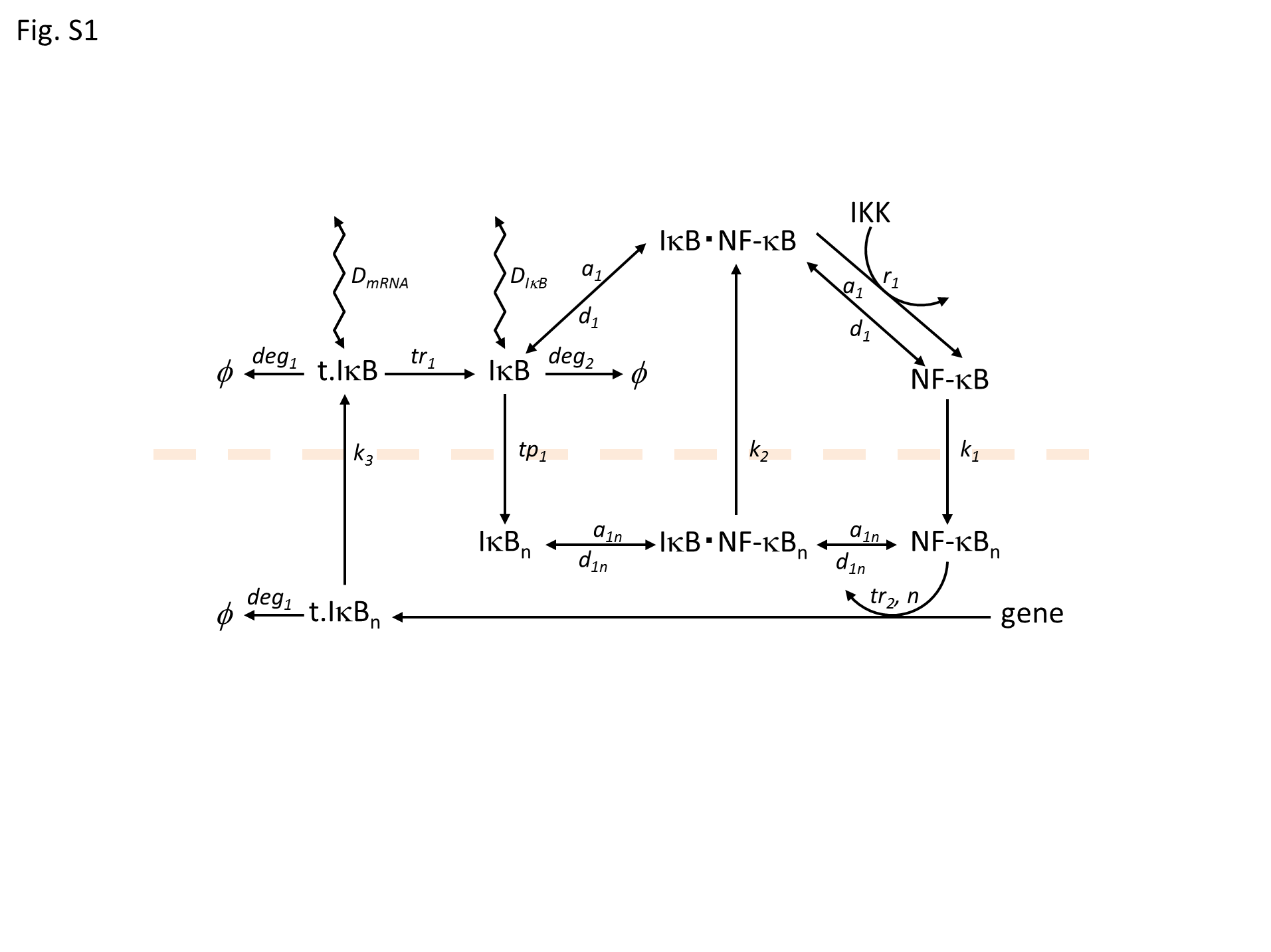

Supplement: S1 Fig — The scheme is identical to that used in a previous report [23]. The diffusion process both for mRNAIκB (t.IκB) and protein IκB are explicitly shown by zigzag lines with bidirectional arrowheads, since these played an important role in the persistency of NF-κBn oscillation (see text). Other species diffused with diffusion coefficients of 10−11 m2/s. (TIF) [file pone.0127633.s001.tif]

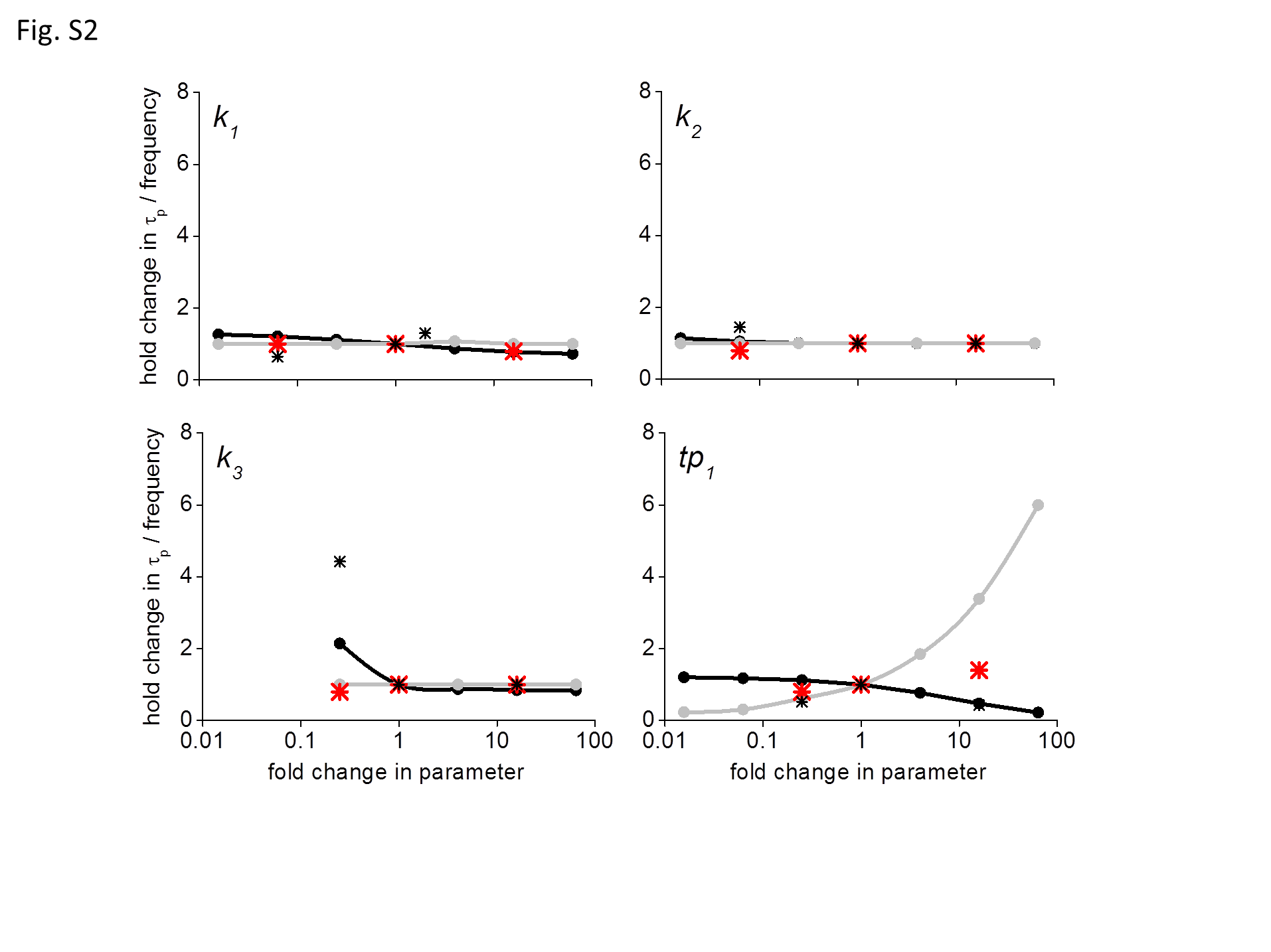

Supplement: S2 Fig — Simulation results of τ p (black asterisks) and frequency (red asterisks) at selected value of each parameter in the 3D model are shown together with those used in the 1D simulations (black and gray circles). Although there were discrepancies between 1D and 3D simulations in k 3 at 0.25-fold decrease and tp 1 at 16-fold increase, the overall propensity of the change agreed between the 3D and 1D simulations. (TIF) [file pone.0127633.s002.tif]

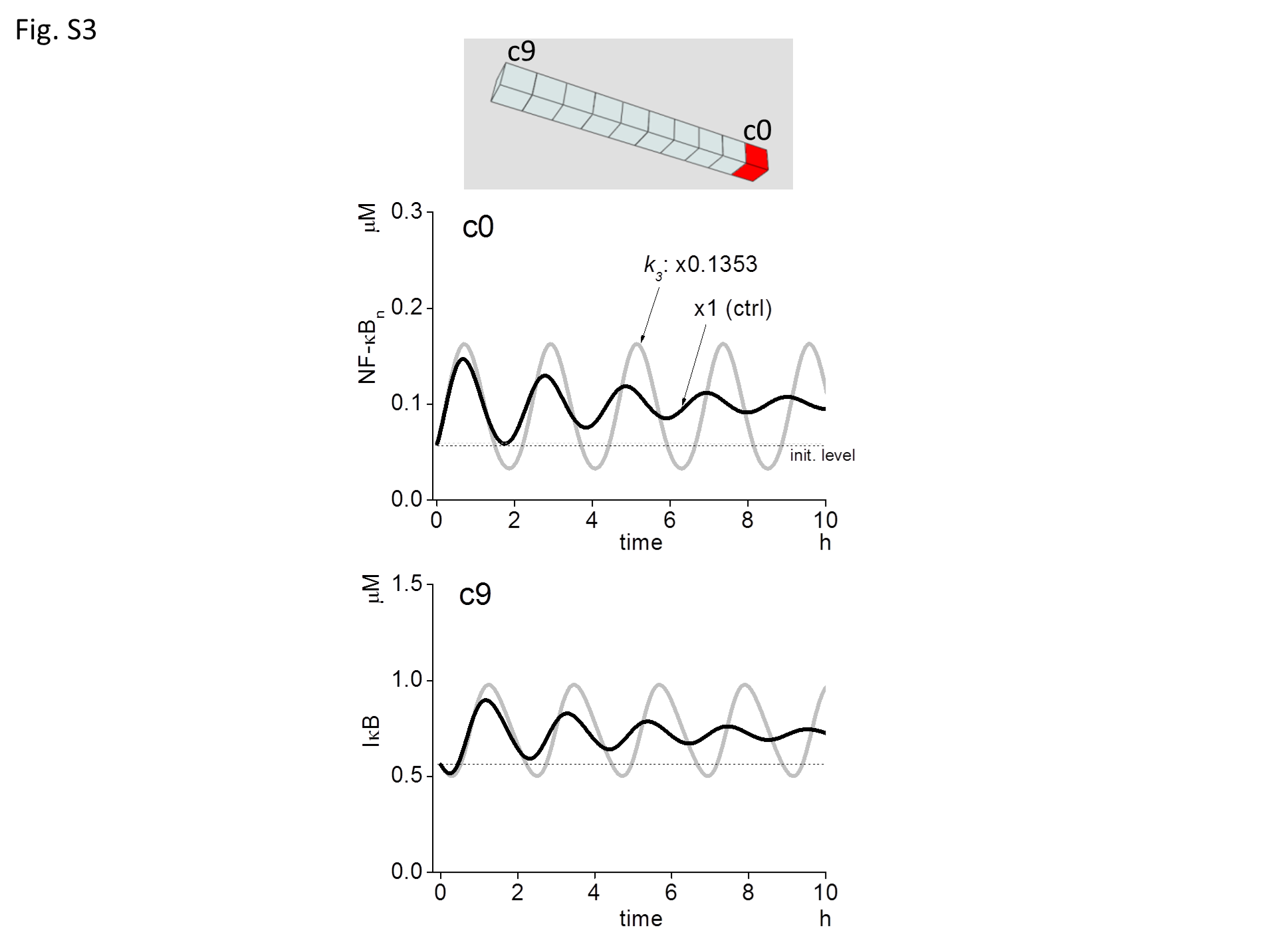

Supplement: S3 Fig — At c0 (nuclear compartment), the NF-κBn concentration at the troughs was larger for the dampened oscillation and smaller for the sustained oscillation at control value of k 3 and 0.1353-fold of the control, respectively (middle panel), similar to the case of diffusion coefficient. However, there was no appreciable difference in the average level of IκB at c9 (most distant cytoplasmic compartment) in both oscillations (bottom panel), which was different from the case of diffusion coefficient. (TIF) [file pone.0127633.s003.tif]

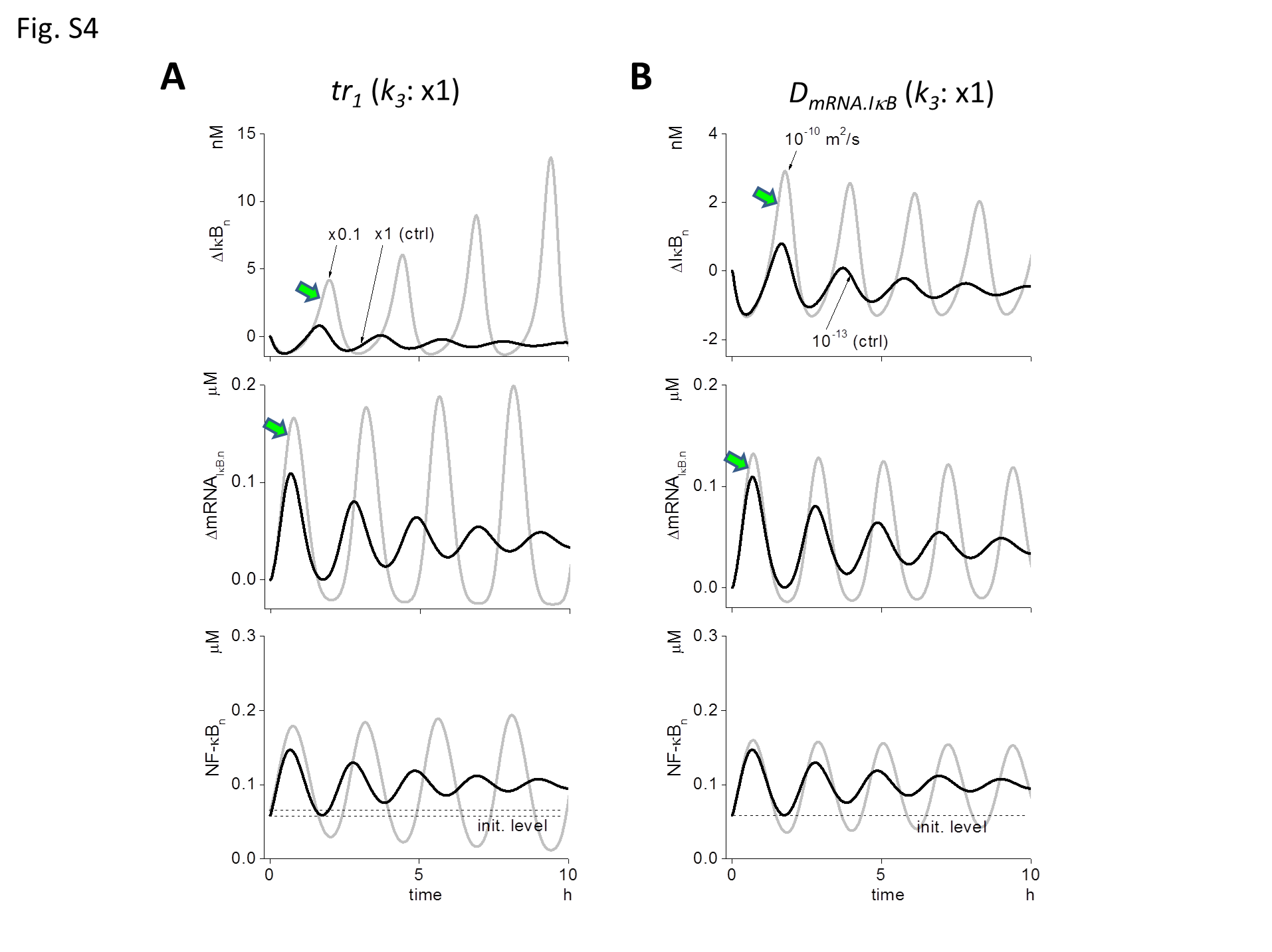

Supplement: S4 Fig — We found the same steep increases in IκBn and mRNAIκB.n (green arrows) caused by the decreasing or increasing tr 1 (A) or D mRNA.IκB (B) at the control level of k 3. The levels of NF-κBn were lower at troughs than initial levels indicating a sufficient “reset” under these conditions, which led to persistent oscillation. (TIF) [file pone.0127633.s004.tif]

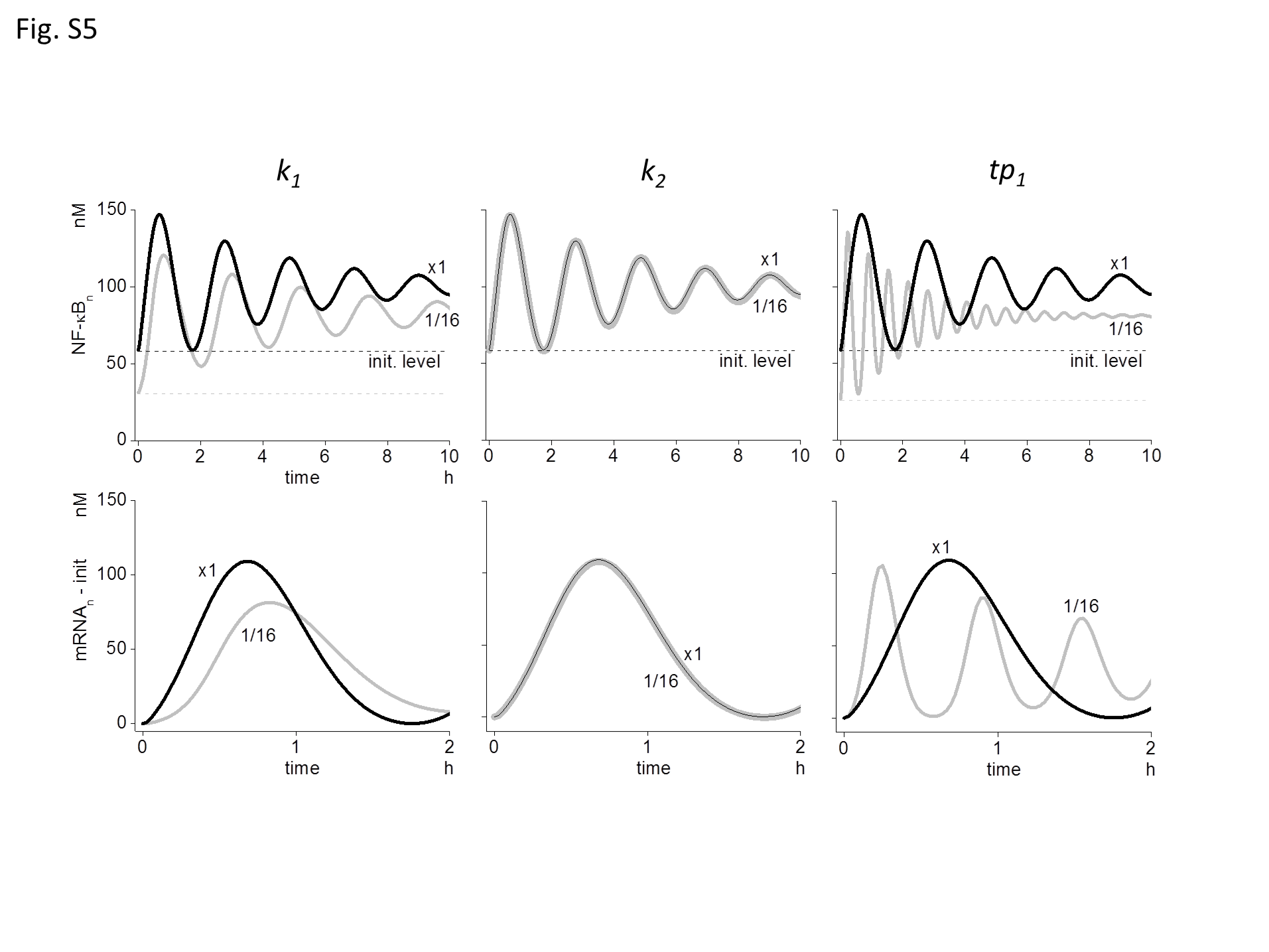

Supplement: S5 Fig — Neither “reset” nor a steep increase in mRNAIkB.n was observed following changes in these parameters. The time course of NF-kB and mRNAIkBn overlapped almost completely after a reduction of k 2 to 1/16 (middle panels). (TIF) [file pone.0127633.s005.tif]

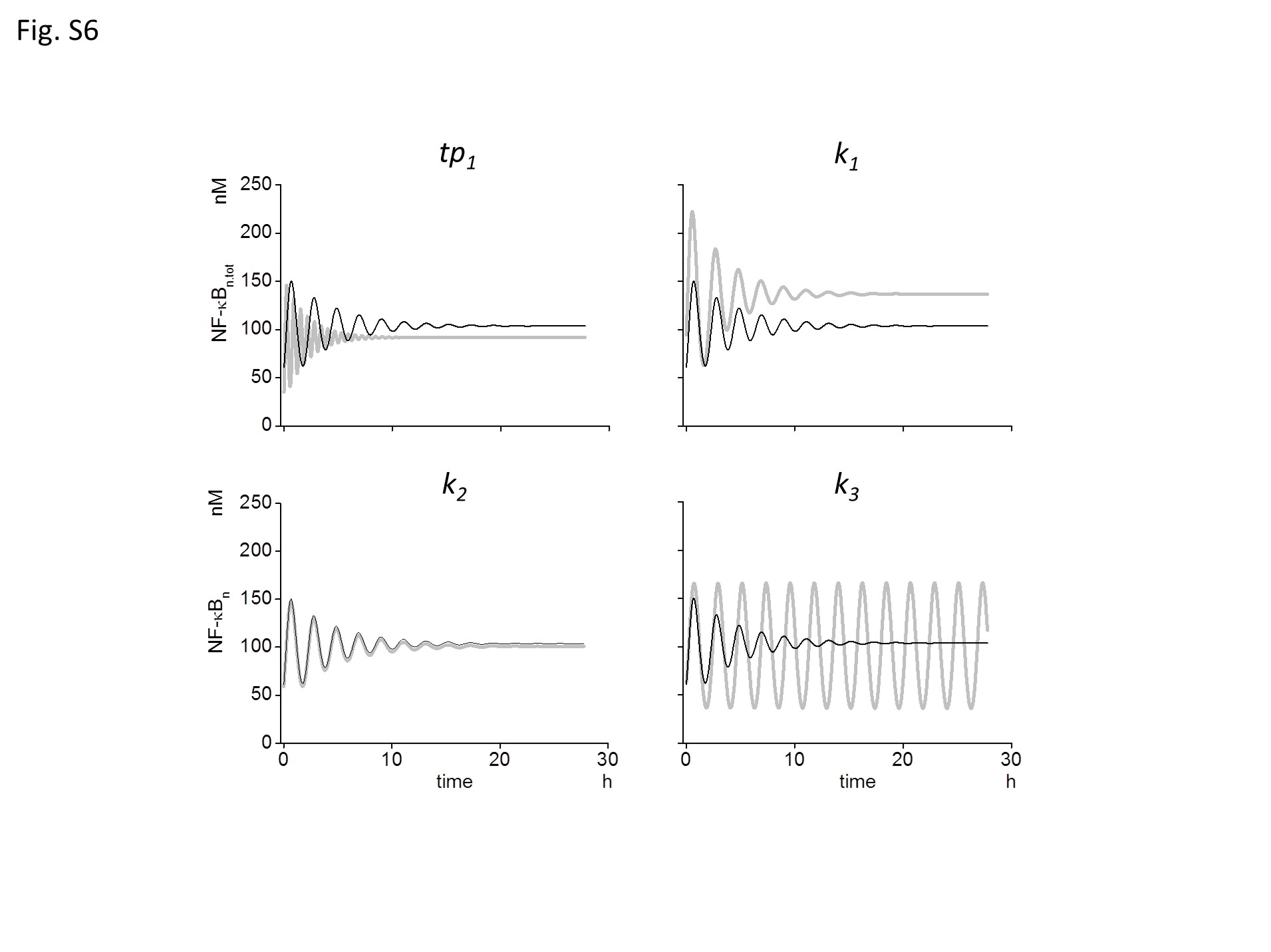

Supplement: S6 Fig — There was virtually no change in the amplitude following changes in tp 1 and k 2, and there was a small change in the amplitude following changes in k 1 and k 3. Thin black and thick gray lines indicate the NF-κBn.tot oscillation under control conditions and at the 16-fold increase (tp 1, k 1, and k 2) or 1/7.4-fold decrease (k 3). (TIF) [file pone.0127633.s006.tif]

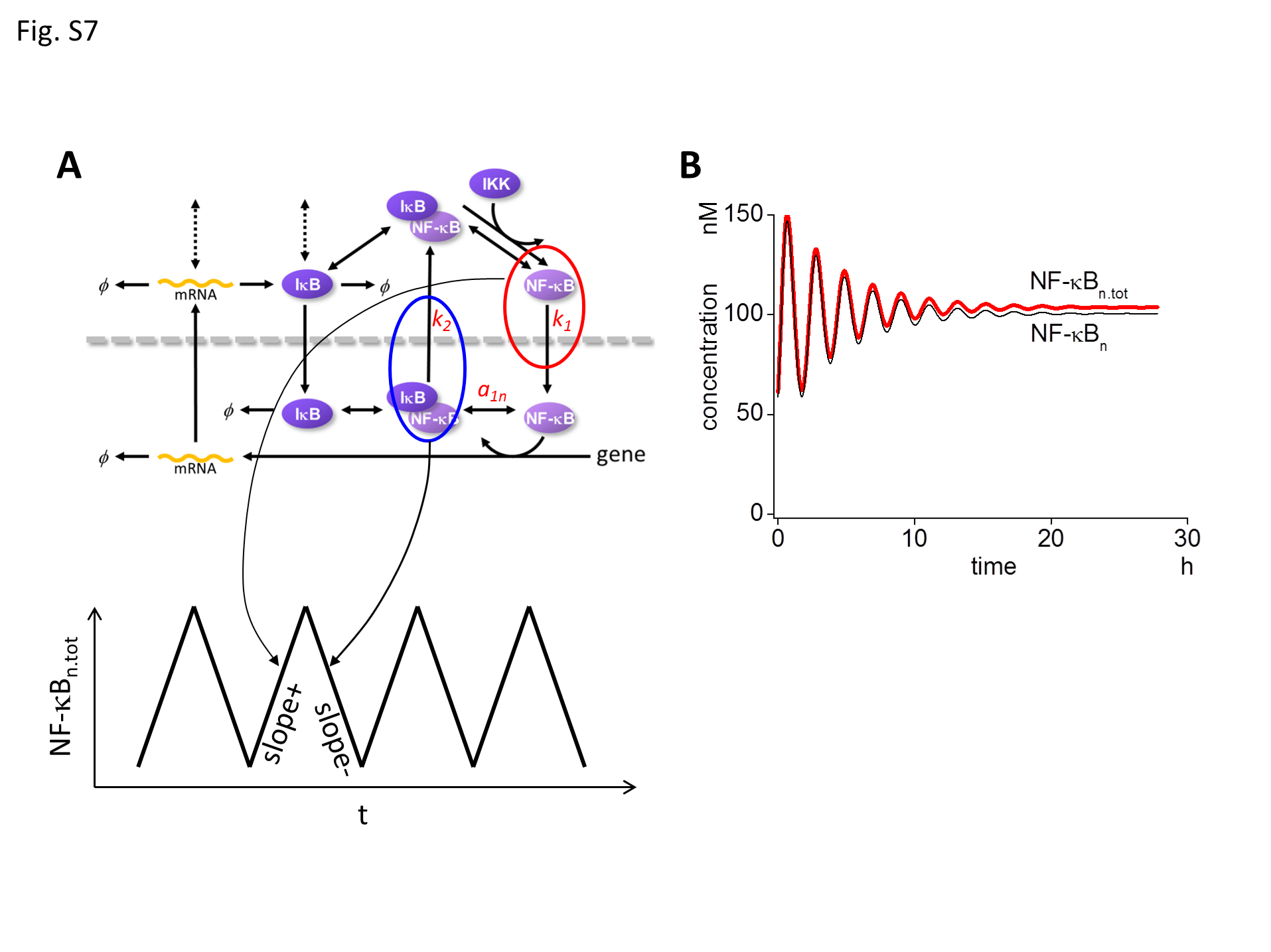

Supplement: S7 Fig — (A) If we approximated the oscillation by a triangular waveform, two slopes (slope+ and slope-) regulated the frequency. Slope+ and slope- were directly calculated by the inward and outward fluxes of NF-κB (k 1*NF-κB) and IκBn:NF-κBn complex (k 2*IκBn:NF-κBn). (B) There was almost no difference between NF-κBn.tot and NF-κBn indicating that we could perform the analyses using NF-κBn instead of NF-κBn.tot. (TIF) [file pone.0127633.s007.tif]

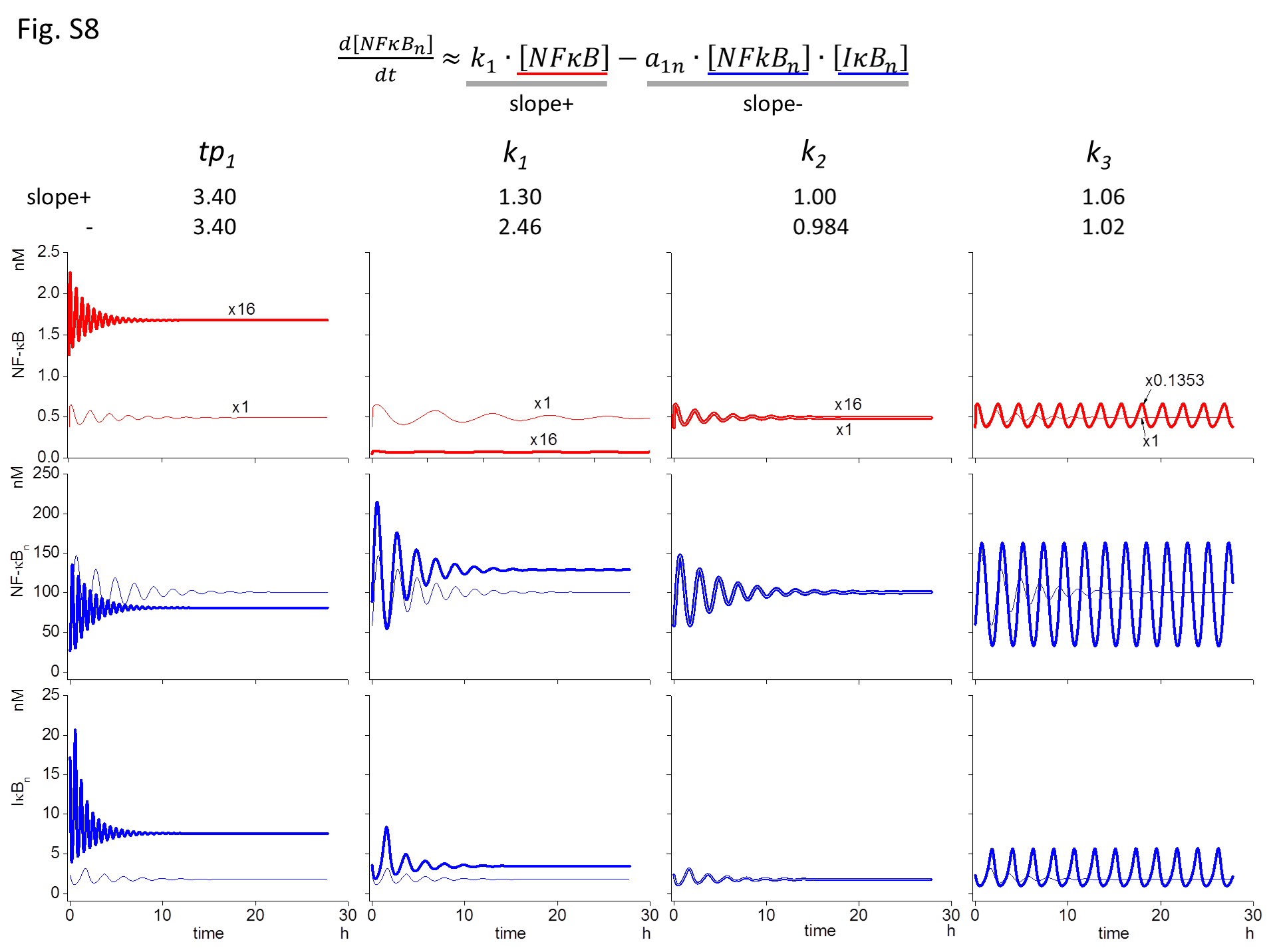

Supplement: S8 Fig — To generate slope+ and slope-, we needed to know the concentrations of NF-κB (top), NF-κBn (middle), and IκBn (bottom). We used average concentrations at equilibrium. Estimated slopes relative to the control values are listed. While a 3.40-fold increase in slope+ and slope- resulted from a 16-fold increase in tp 1, virtually no change occurred following a 16-fold increase in k 2 and a 0.1353-fold decrease in k 3. Increase in k 1 generated marginal changes. (TIF) [file pone.0127633.s008.tif]

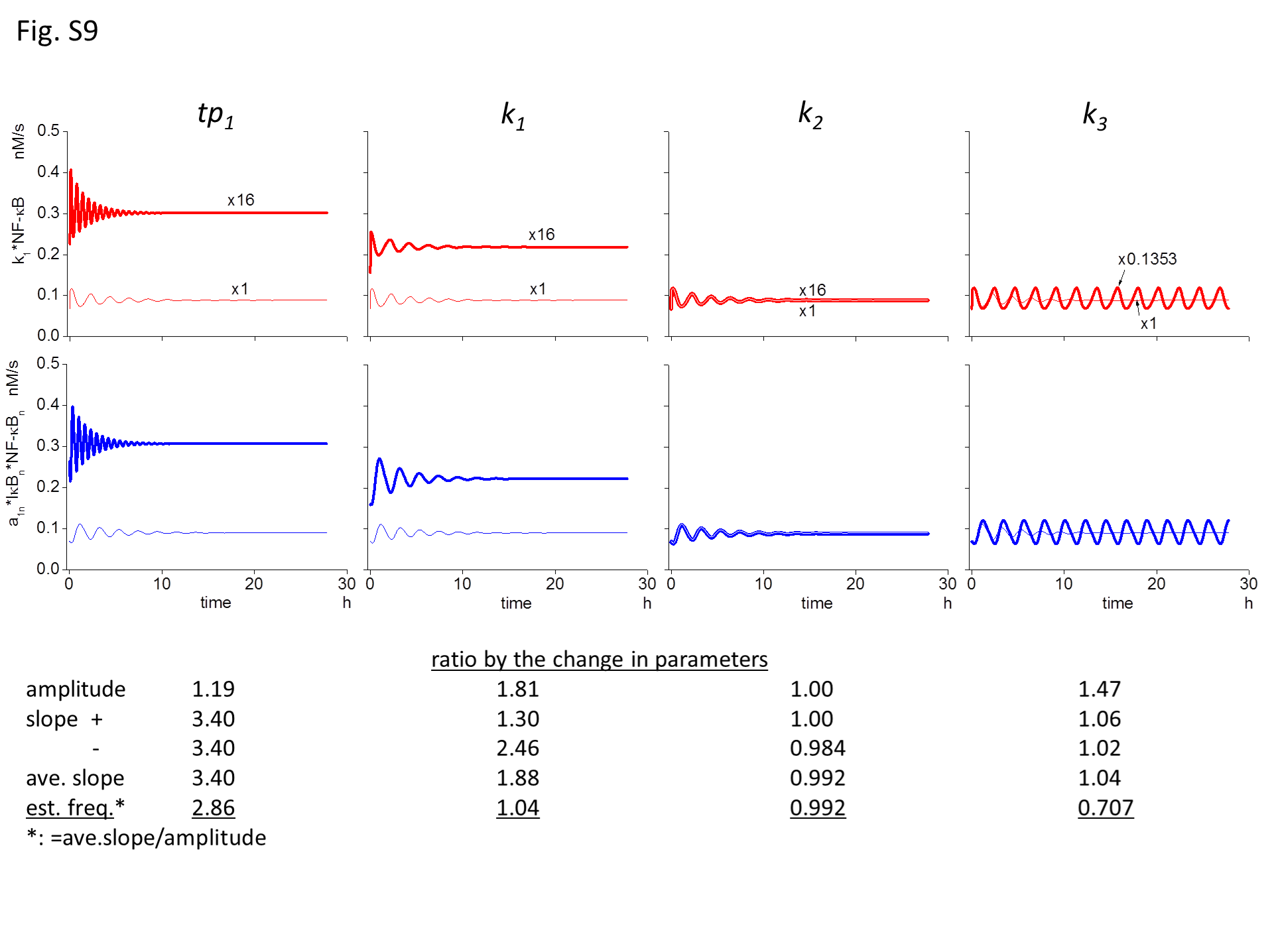

Supplement: S9 Fig — Red and blue lines are slope+ and slope- under control (thin lines) and changed conditions (thick lines) designated in each panel. Estimated changes in the frequency (est.freq.) were calculated by the change in the amplitude and the average slope by Eq 2. Only change in tp 1 resulted in an appreciable change in the frequency. (TIF) [file pone.0127633.s009.tif]

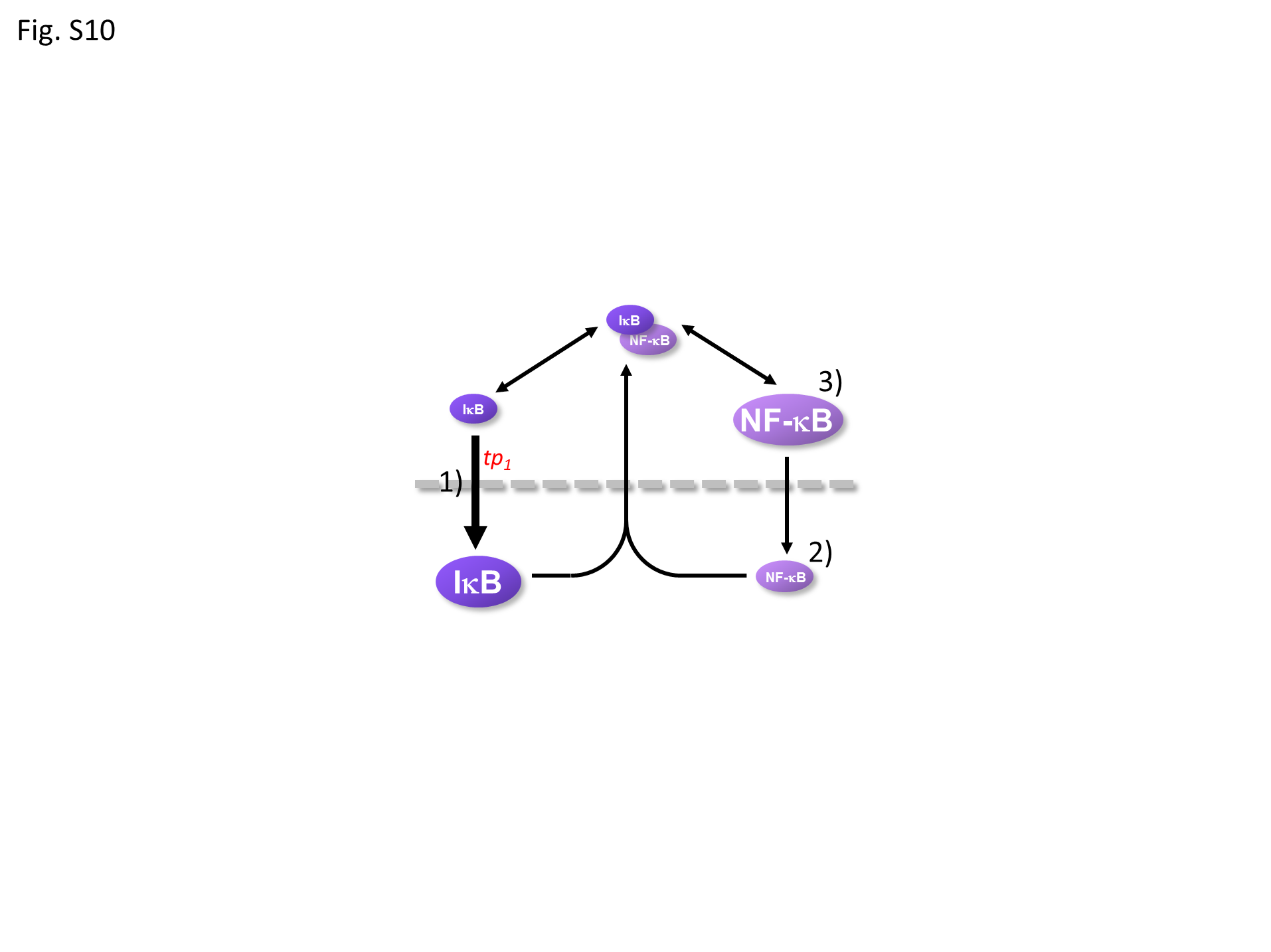

Supplement: S10 Fig — 1) Larger tp 1 increased the inward flux of IκB resulting in the reduction of cytoplasmic IκB. 2) This increased IκBn led to the reduction of NF-κBn due to the increase in the efflux of NF-κBn. 3) Because of the increase in the NF-κBn efflux, the cytoplasmic NF-κB increased. Thus, the equilibrium changed to a state of greater cytoplasmic NF-κB. (TIF) [file pone.0127633.s010.tif]

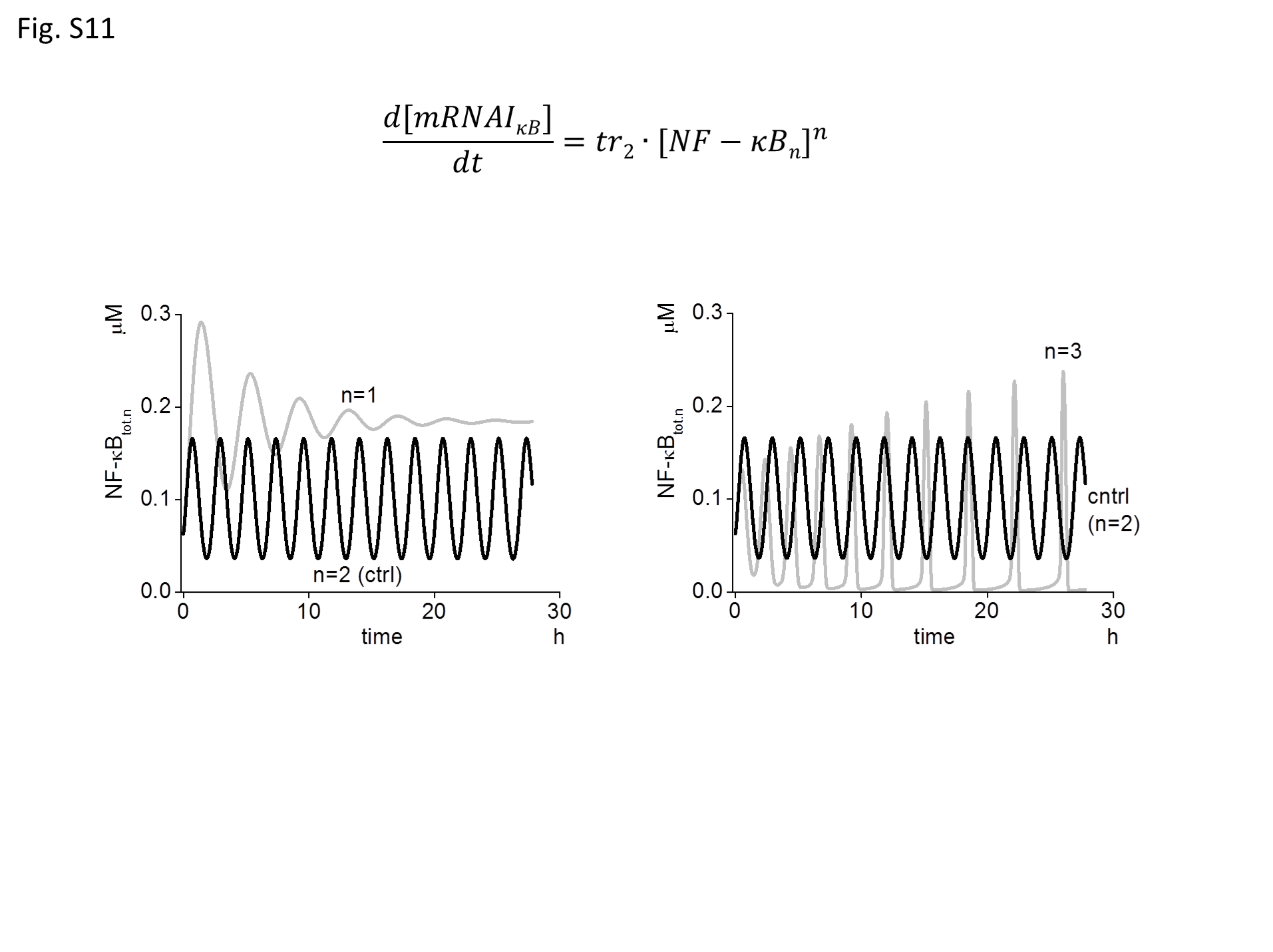

Supplement: S11 Fig — Transcription of mRNAIκB was calculated by the equation shown above. There were two parameters controlling the transcription, tr 2 and n. Among them, n described the nonlinearity of the transcription in relation to the concentration of NF-κBn. n = 2 at the control condition, assuming that the binding of two NF-κB molecules to the κB site of genes was required for their regulation. If n was set to 1 or 3, the sustained oscillation at 0.1353-fold decreased k 3 resulted in a dampened or an inflating oscillation (left and right panels, respectively). (TIF) [file pone.0127633.s011.tif]

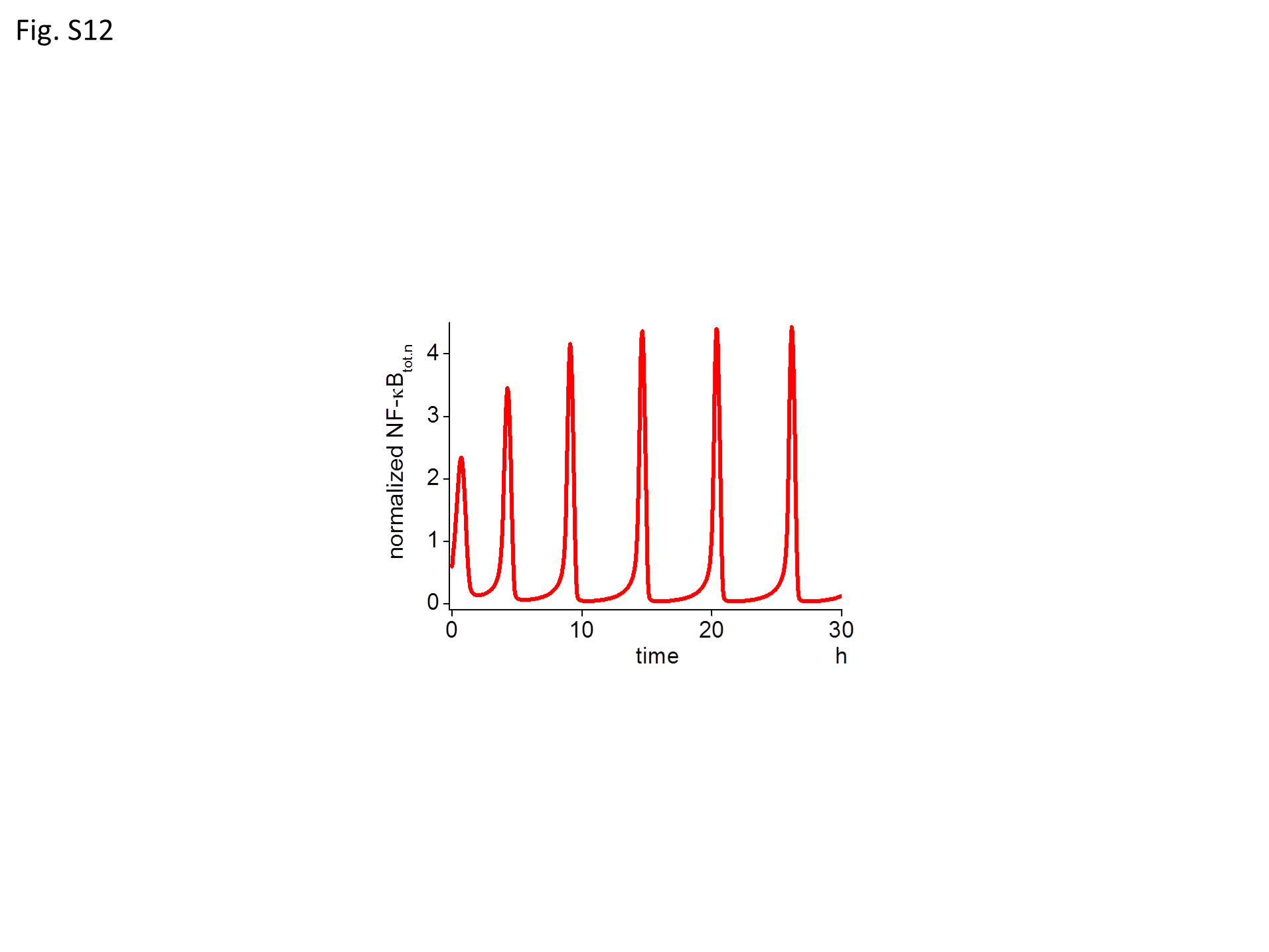

Supplement: S12 Fig — When the rate of IκB kinase (IKK) degradation was set to 0 as in the 1D model, inflating oscillation was observed in the 3D model as well. (TIF) [file pone.0127633.s012.tif]
